# Supplementary material for: Impact of fatigue as the primary determinant of functional limitations among patients with post-COVID-19 syndrome: a cross-sectional observational study
Source: BMJ Open. 2023 Jun 5;13(6):e069217. doi: 10.1136/bmjopen-2022-069217 (PMC10335413; doi:10.1136/bmjopen-2022-069217)

## Appendix

**Appendix 1:** Mean scores for the overall WSAS score and individual WSAS domains

| WSAS Domain | N | Mean (SD) | Range and threshold values |
|---|---|---|---|
| WSAS overall score | 2627 | 20.6 (9.9) | Range:0-40<br><10: subclinical<br>10 – 19: significant<br>**>20: Moderately severe** |
| Ability to work* | 2621 | 4.6 (2.4) | Subscale range: 0-8<br>0: not at all affected to<br>8: very severely affected |
| Home management | 2627 | 4.2 (2.2) | |
| Social leisure activities | 2627 | 4.0 (2.2) | |
| Private leisure activities | 2627 | 4.7 (2.3) | |
| Close relationships | 2627 | 3.0 (2.4) | |

* Reduced number of completed answers as patients who had retired or chose not to work did not need to answer this question.

Appendix 2: EQ-5D-5L frequencies and proportions reported by dimension and level

| | Mobility<br>n (%) | Self-care<br>n (%) | Usual activities<br>n (%) | Pain / discomfort<br>n (%) | Anxiety / depression<br>n (%) |
|---|---|---|---|---|---|
| **Level 1**<br>(No problems) | 712(26.9) | 318(12.0) | 959(36.3) | 101(3.8) | 134(5.1) |
| **Level 2**<br>(Slight problems) | 795(30.1) | 1702(64.4) | 250(9.5) | 983(37.2) | 701(26.5) |
| **Level 3**<br>(Moderate problems) | 309(11.7) | 98(3.7) | 506(19.1) | 358(13.5) | 675(25.5) |
| **Level 4**<br>(Severe problems) | 810(30.6) | 511(19.3) | 759(28.7) | 373(14.1) | 267(10.1) |
| **Level 5**<br>(Extreme problems / unable to do) | 17(0.6) | 14(0.5) | 169(6.4) | 828(31.3) | 866(32.8) |
| Total | 2643(100) | 2643(100) | 2643(100) | 2643(100) | 2643(100) |

**Appendix 3:** Working days lost due to Post-COVID syndrome in 28 days prior to completion of Service Use Questionnaire

| | |
|---|---|
| Number completed Service Use questionnaire | 2600 |
| Number (%) who lost 1 or more days from work | 1321 (50.8) |
| Mean number of working days lost (SD)* | 13.8 (10.7) |
| Median number of working days lost (IQR)* | 10 (4 to 28) |

* in those who lost 1 or more days off work

**Appendix 4:** EQ-5D index score multivariable model for different patient characteristics and PROM scores (N=2405)

| Patient Characteristics | | Model coefficients (95% CI) | p-value | Change in R-squared * [Full model R-sq=0.573) | Standardised effect size |
|---|---|---|---|---|---|
| Age | 18 to 29 | Reference | | | |
| | 30 to 39 | -0.02 (-0.05, 0.01) | 0.219 | | |
| | 40 to 49 | -0.03 (-0.06, -0.01) | 0.009 | | |
| | 50 to 59 | -0.03 (-0.06, -0.01) | 0.018 | | |
| | 60 to 69 | -0.06 (-0.09, -0.03) | <0.0001 | | |
| | 70 and over | -0.07 (-0.12, -0.02) | 0.005 | | |
| Gender | Male | Reference | | | |
| | Female | 0.00 (-0.01, 0.02) | 0.786 | | |
| Educational | No education | Reference | | | |

| | | | | | |
|---|---|---|---|---|---|
| level | School leaver (NVQ 1-2) | 0.00 (-0.04, 0.04) | 0.948 | | |
| | A-Level (NVQ-3) | 0.02 (-0.02, 0.05) | 0.389 | | |
| | Degree (NVQ-4) | 0.01 (-0.02, 0.05) | 0.464 | | |
| | Postgraduate degree (NVQ-5) | 0.02 (-0.01, 0.06) | 0.210 | | |
| Ethnicity | White | Reference | | | |
| | Non-white | -0.02 (-0.04, 0.00) | 0.073 | | |
| IMD Quintile | 1 (most deprived) | Reference | | | |
| | 2 | 0.02 (0.00, 0.05) | 0.059 | | |
| | 3 | 0.03 (0.00, 0.05) | 0.025 | | |
| | 4 | 0.05 (0.02, 0.07) | <0.0001 | | |
| | 5 (least deprived) | 0.03 (0.01, 0.06) | 0.008 | | |
| PROMs | FACIT-Fatigue (reversed scale) | -0.01 (-0.01, -0.01) | <0.0001 | 0.048 | -0.080 |
| | PHQ-8 | -0.01 (-0.01, -0.01) | <0.0001 | 0.010 | -0.044 |

| | | | | | |
|---|---|---|---|---|---|
| | GAD-7 | -0.01 (-0.01, -0.01) | <0.0001 | 0.018 | -0.051 |
| | MRC Dyspnoea Scale: Grade 1 | Reference | | 0.032 | |
| | MRC Dyspnoea Scale: Grade 2 | 0.02 (0.00, 0.04) | 0.108 | | 0.010 |
| | MRC Dyspnoea Scale: Grade 3 | -0.02 (-0.04, 0.01) | 0.191 | | -0.009 |
| | MRC Dyspnoea Scale: Grade 4 | -0.08 (-0.11, -0.05) | <0.0001 | | -0.030 |
| | MRC Dyspnoea Scale: Grade 5 | -0.25 (-0.30, -0.20) | <0.0001 | | -0.045 |
| | Dyspnoea-12 | 0.00 (0.00, 0.00) | <0.0001 | 0.003 | -0.020 |

* Reduction in R-squared value when variable is removed from the final model. Overall model has R-squared value of 0.573

Appendix Figure 1: Frequency distribution of the first reported (baseline) WSAS

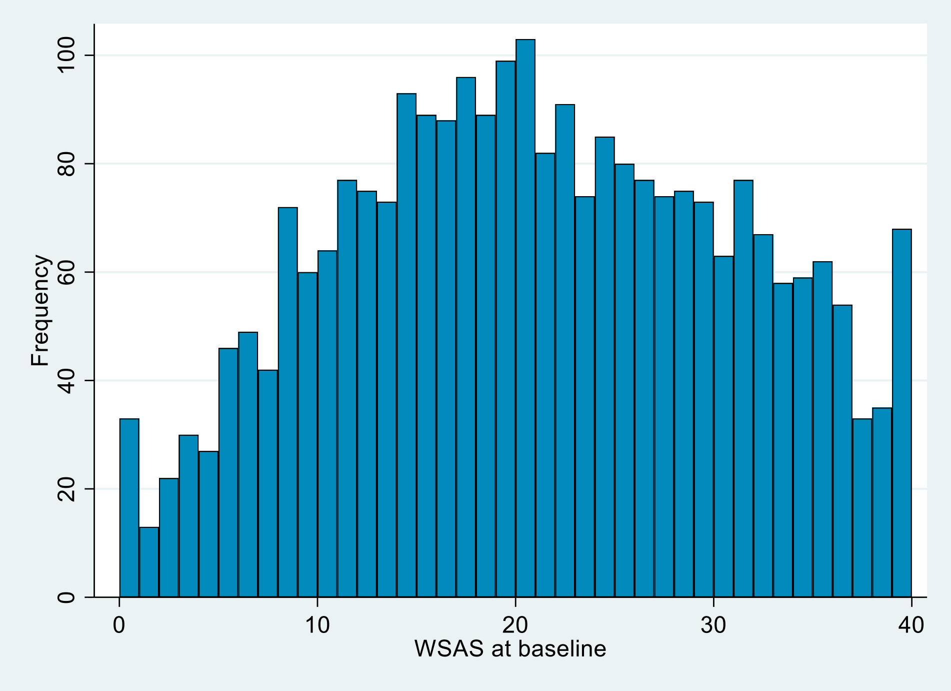


Appendix Figure 2: Frequency distribution of the first reported (baseline) EQ-5D Index Score

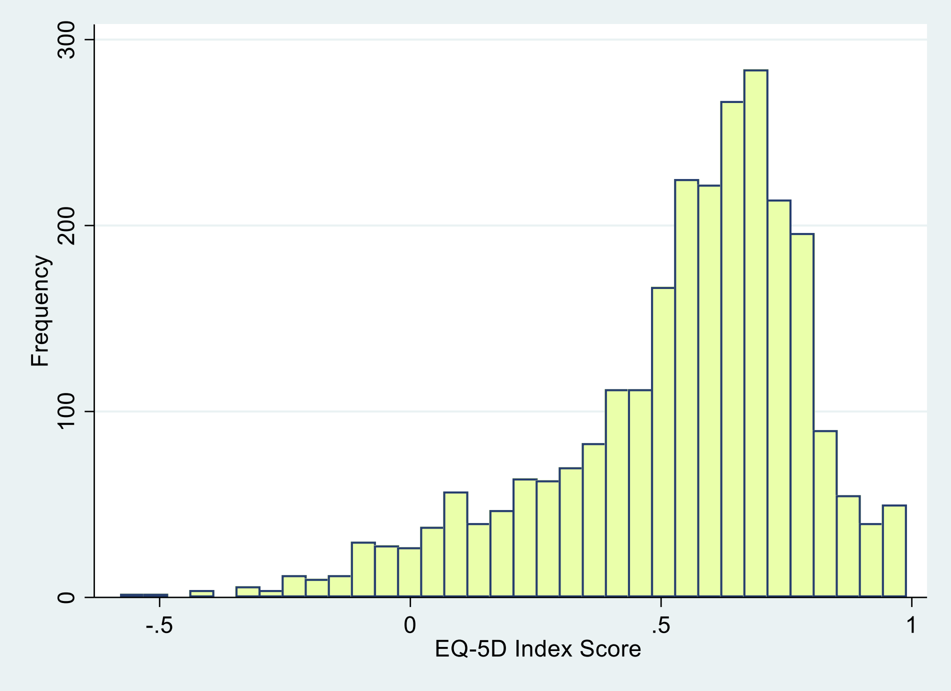

Supplement: Supplementary data [file bmjopen-2022-069217supp001.pdf]
