# Supplementary material for: Impact of fatigue as the primary determinant of functional limitations among patients with post-COVID-19 syndrome: a cross-sectional observational study
Source: BMJ Open. 2023 Jun 5;13(6):e069217. doi: 10.1136/bmjopen-2022-069217 (PMC10335413; doi:10.1136/bmjopen-2022-069217)
Supplement: Author's manuscript [file bmjopen-2022-069217.draft_revisions.pdf]

BMJ Open is committed to open peer review. As part of this commitment we make the peer review history of every article we publish publicly available.

When an article is published we post the peer reviewers' comments and the authors' responses online. We also post the versions of the paper that were used during peer review. These are the versions that the peer review comments apply to.

The versions of the paper that follow are the versions that were submitted during the peer review process. They are not the versions of record or the final published versions. They should not be cited or distributed as the published version of this manuscript.

BMJ Open is an open access journal and the full, final, typeset and author-corrected version of record of the manuscript is available on our site with no access controls, subscription charges or pay-per-view fees (http://bmjopen.bmj.com).

If you have any questions on BMJ Open's open peer review process please email info.bmjopen@bmj.com

BMJ Open

The impact of fatigue as the primary determinant of functional limitations amongst patients with Post-COVID syndrome: a cross-sectional observational study

Journal:	*BMJ Open*
Manuscript ID	bmjopen-2022-069217
Article Type:	Original research
Date Submitted by the Author:	02-Nov-2022

Complete List of Authors:	Walker, Sarah; University of Exeter, The Department of Health and Community Sciences (Medical School)<br>Goodfellow, Henry; University College London, Department of Primary Care and Population Health<br>Pookarnjanamorakot, Patra; Whittington Health NHS Trust, General Medicine<br>Murray, Elizabeth; University College London, Primary Care and Population Health<br>Bindman, Julia; University College London, Research Department of Primary Care and Population Health<br>Blandford, Ann; University College London, UCLIC, Dept of Computer Science<br>Bradbury, Katherine; University of Southampton, Psychology<br>Cooper, Belinda; University College London, Research Department of Primary Care and Population Health<br>Hamilton, Fiona; University College London, Research Department of Primary Care and Population Health<br>Hurst, John; University College London, UCL Respiratory<br>Hylton, Hannah; Barts Health NHS Trust, Department of Respiratory Medicine<br>Linke, Stuart; University College London, Research Department of Primary Care and Population Health<br>Pfeffer, Paul; Barts Health NHS Trust, Department of Respiratory Medicine<br>Ricketts, William; Barts Health NHS Trust, Respiratory Medicine<br>Robson, Chris; Living With<br>Stevenson, Fiona; University College London, Research Department of Primary Care and Population Health<br>Sunkersing, David; University College London, Research Department of Primary Care and Population Health<br>Wang, Jiunn; University College London, Department of Applied Health Research<br>Gomes, Manuel; University College London, Department for Applied Health Research<br>Henley, William; University of Exeter, The Department of Health and Community Sciences (Medical School)<br>Collaboration, LivingWith Covid Recovery ; University College London

Keywords:	COVID-19, Public health < INFECTIOUS DISEASES, MENTAL HEALTH,

	PRIMARY CARE, Anxiety disorders < PSYCHIATRY, Depression & mood disorders < PSYCHIATRY

SCHOLARONE™
Manuscripts

I, the Submitting Author has the right to grant and does grant on behalf of all authors of the Work (as defined in the below author licence), an exclusive licence and/or a non-exclusive licence for contributions from authors who are: i) UK Crown employees; ii) where BMJ has agreed a CC-BY licence shall apply, and/or iii) in accordance with the terms applicable for US Federal Government officers or employees acting as part of their official duties; on a worldwide, perpetual, irrevocable, royalty-free basis to BMJ Publishing Group Ltd ("BMJ") its licensees and where the relevant Journal is co-owned by BMJ to the co-owners of the Journal, to publish the Work in this journal and any other BMJ products and to exploit all rights, as set out in our licence.

The Submitting Author accepts and understands that any supply made under these terms is made by BMJ to the Submitting Author unless you are acting as an employee on behalf of your employer or a postgraduate student of an affiliated institution which is paying any applicable article publishing charge ("APC") for Open Access articles. Where the Submitting Author wishes to make the Work available on an Open Access basis (and intends to pay the relevant APC), the terms of reuse of such Open Access shall be governed by a Creative Commons licence – details of these licences and which Creative Commons licence will apply to this Work are set out in our licence referred to above.

Other than as permitted in any relevant BMJ Author's Self Archiving Policies, I confirm this Work has not been accepted for publication elsewhere, is not being considered for publication elsewhere and does not duplicate material already published. I confirm all authors consent to publication of this Work and authorise the granting of this licence.

For peer review only

Title The impact of fatigue as the primary determinant of functional limitations amongst patients with Post-COVID syndrome: a cross-sectional observational study

Corresponding Author:

Professor William Henley, The Department of Health and Community Sciences (Medical School), University of Exeter, St Luke's Campus, Exeter, England, EX1 2LU

Email: W.E.Henley@exeter.ac.uk

Authors

Dr Sarah Walker, The Department of Health and Community Sciences (Medical School), University of Exeter, St Luke's Campus, Exeter, UK, EX1 2LU.

Henry Goodfellow, Research Department of Primary Care and Population Health, University College London, London, UK

Patra Pookarnjanamorakot, General Medicine, Whittington Health NHS Trust, London, UK

Elizabeth Murray, Research Department of Primary Care and Population Health, University College London, London, UK

Julia Bindman, Research Department of Primary Care and Population Health, University College London, London, UK

Ann Blandford, UCLIC, Dept of Computer Science, UCL, London WC1E 6BT, UK.

Katherine Bradbury, Psychology, University of Southampton, Southampton, UK

Belinda Cooper, Research Department of Primary Care and Population Health, University College London, London, UK

Fiona L Hamilton, Research Department of Primary Care and Population Health, University College London, London, UK

John R Hurst, UCL Respiratory, University College London, London, UK

Hannah Hylton, Department of Respiratory Medicine, Barts Health NHS Trust, London, UK

Stuart Linke, Camden and Islington Mental Health Trust and Research Department of Primary Care and Population Health, University College London, London, UK

Paul E Pfeffer, Department of Respiratory Medicine, Barts Health NHS Trust, London, UK and Barts and The London School of Medicine and Dentistry, Queen Mary University of London, London, UK.

William Ricketts, Department of Respiratory Medicine, Barts Health NHS Trust, London, UK

Chris Robson, Living With, London, UK

Fiona A Stevenson, Research Department of Primary Care and Population Health, University College London, London, UK

For peer review only

David Sunkersing, Research Department of Primary Care and Population Health, University College London, London, UK

Jiunn Wang, Department of Applied Health Research, University College London, London, UK

Manuel Gomes, Department for Applied Health Research, University College London, London, UK

William Henley, The Department of Health and Community Sciences (Medical School), University of Exeter, St Luke's Campus, Exeter, UK, EX1 2LU.

On behalf of the Living With Covid Recovery Collaboration

Word count 4626

Keywords

Fatigue, functional limitation, Long-COVID, Post-COVID, COVID-19, WSAS, SARS-CoV-2, EQ-5D, HRQoL

For peer review only

ABSTRACT

Objectives

To describe self-reported characteristics and symptoms of treatment-seeking Post-COVID Syndrome (PCS) patients. To assess the impact of symptoms on health-related quality of life and patients' ability to work and undertake activities of daily living.

Design

Cross-sectional single-arm service evaluation of real-time user data.

Setting

31 Post-COVID clinics in the UK.

Participants

3,754 adults diagnosed with PCS in primary or secondary care, deemed suitable for rehabilitation.

Intervention

Patients using the Living With Covid Recovery (LWCR) Digital Health Intervention (DHI) registered between 30/11/20 and 23/03/22.

Primary and secondary outcome measures

The primary outcome was the baseline Work and Social Adjustment Scale (WSAS). WSAS measures the functional limitations of the patient; scores ≥20 indicate moderately severe limitations. Other symptoms explored included fatigue (FACIT-F), depression (PHQ-8), anxiety (GAD-7), breathlessness (MRC Dyspnoea Scale and Dyspnoea-12), cognitive impairment (PDQ-5) and health-related quality of life (EQ-5D). Symptoms and demographic characteristics associated with more severe functional limitations were identified using logistic regression analysis.

Results

3541 (94%) patients were of working age (18-65); mean age (SD) 48 (12) years; 1282 (71%) were female and 89% were White. 51% reported losing ≥1 days from work in the previous 4 weeks; 20% reported being unable to work at all. Mean WSAS score at baseline was 21 (SD 10) with 53% scoring ≥20. Factors associated with WSAS scores ≥20 were high levels of fatigue, depression and cognitive impairment. Fatigue was found to be the main symptom contributing to a high WSAS score.

Conclusions

A high proportion of this PCS treatment-seeking population was of working age with over half reporting moderately severe or worse functional limitation. There were substantial impacts on ability to work and activities of daily living in people with PCS. Clinical care and rehabilitation should address the management of fatigue as the dominant symptom explaining variation in functionality.

(299 words)

For peer review only

Summary Box

Section 1: What is already known on this topic

Post-COVID syndrome (PCS) is a complex condition with prolonged heterogeneous symptoms. There have been various estimates on the number of patients with acute COVID-19 that go on to develop PCS, ranging from 3.0% to 14.1%. Most evidence on PCS characteristics comes from studies of people previously hospitalised with COVID-19. An urgent need has been identified to better understand the symptoms and impact of PCS in patients attending primary care or community clinics. This will aid the design and adaptation of existing services for PCS patients.

Section 2: What this study adds

This is the first large-scale study of Post-COVID syndrome symptoms and functional limitations in a treatment-seeking population in the UK.

More than half of this population is experiencing moderately severe or worse functional impairment. This has a substantial impact on ability to work and day-to-day living of the national workforce.

Fatigue is the dominant symptom driving variation in impairment and should form a target for clinical care and design of rehabilitation strategies.

Targeting limiting resources to effectively addressing functional limitations from Post-COVID syndrome has important implications for health service management and will support the continued recovery of the economy.

INTRODUCTION

Post-COVID Syndrome (PCS), or "Long-COVID", is defined by National Institute for Health & Care Research (NIHR) and the World Health Organization (WHO) as the signs and symptoms of the disease that continue for more than 12 weeks after the initial acute covid infection. [1] It is causing increasing concern due to the potential number of patients infected and the associated morbidity caused by the symptoms.

As of the 2nd August 2022, there have been over 577 million cases of COVID-19 worldwide. [2] There have been various estimates on the number of patients with acute COVID-19 that go on to develop PCS, ranging from 3.0% to 14.1% [1 3-6] with over 1.4 million people in the UK reporting PCS symptoms as of July 2022. [6] The symptoms of PCS include fatigue, breathlessness, brain fog, anosmia, and mental health problems. These symptoms can cause debilitating functional and psychological limitations [3 7] and have been shown to persist for up to two years. [1 3 6 8-10] This has led to many people with PCS being unable to work or care for others for a prolonged period. [7] The potential impact of PCS on national health services,

For peer review only - http://bmjopen.bmj.com/site/about/guidelines.xhtml

economies and population health is attracting international attention as the associated morbidity and economic effects become clearer. [5 11-17]

The UK National Health Service (NHS) has set up Post-COVID Assessment Clinics to provide care for the large number of patients with PCS .[6 18] In the absence of pharmacotherapies shown to be effective for this condition, management of people with PCS has to date focused on self-management education and rehabilitation programmes. These clinics provide specialist rehabilitation from a range of health care professionals including respiratory specialist doctors, GPs, Physiotherapists, Occupational Therapists and Psychologists. Over 30 of these clinics were augmented with a bespoke Digital Health Intervention (DHI), called Living With Covid Recovery, to enable remote rehabilitation for PCS patients during the COVID-19 pandemic. Internationally, despite the growing number of PCS patients, the strategies to combat PCS are at their early stages with no standard rehabilitation pathway. [11-14] As the pandemic continues, PCS will continue to add significant workload for health services beyond acute COVID-19 care.[19]

This study is the first to present the baseline symptoms and functional impairment from a treatment-seeking PCS population across multiple centres and to estimate the contribution of different patient-reported symptoms to impairment. These data will help clinicians and policy makers plan appropriate services.

METHODS

Design and setting

Cross-sectional observational study of patients using the Living With Covid Recovery Digital Health Intervention as part of their assessment and treatment in 31 self-selecting specialised Post-COVID clinics in England and Wales.

Intervention

Living With Covid Recovery (LWCR) is a bespoke Digital Health Intervention (DHI), designed to be part of Post-COVID Clinics. The LWCR DHI was designed by a multi-disciplinary team of clinicians, Patient and Public Involvement (PPI), academics and industry partners.[20] The product was first launched in a clinical setting in August 2020 and since then has been updated 8 times. It contains 13 (11 validated) patient-reported outcome measures (PROMs) in the form of validated questionnaires completed by patients as part of their clinical care. Seven related to symptoms and one related to each of patient demographics, functional ability, quality of life and health service use. More details are provided in the 'Patient Reported Outcome Measures (PROMs)' section below and in the study protocol. The WSAS questionnaire was introduced in February 2021 and the Demographic questionnaire in April 2021. Development followed the principles of human computer interaction agile development, with updates to the DHI based on feedback from healthcare practitioners and our PPI group. All data collected in the LWCR product were pseudo-anonymised, using a unique patient ID number and stored in Metabase (www.metabase.com).

Population

Patients included in this study were those who had registered to use the LWCR DHI as part of the clinical care provided in a Post-COVID Syndrome NHS community clinic in England and

For peer review only - http://bmjopen.bmj.com/site/about/guidelines.xhtml

Wales. Patients are referred to these clinics from Primary or Secondary Care after having experienced Post-COVID symptoms for 12 weeks or more.

Eligible patients were identified as being suitable for remote rehabilitation service by the clinic if they were aged 18 or over, had access to a smart phone device, were considered likely to benefit from the intervention, fit for rehabilitation and were able to read English. Patients registered on the LWCR DHI between 30/11/20 and 23/03/22.

Outcomes

Primary Outcome

The Work and Social Adjustment Scale (WSAS) was the primary outcome measure for this study. WSAS is a validated questionnaire for functional impairment[21]. Scores range between 0 and 40, with scores of 20 or more indicating moderately severe or worse impairment on daily functioning. [21] The WSAS contains 5 equally weighted component scores (range 0 to 8), relating to impairments across the following domains:

1) Ability to work
2) Home management
3) Social leisure activities
4) Private leisure activities
5) Close relationships

Additionally, there is a further question to identify those individuals who are either retired or have chosen not to work.

Secondary Outcome

The secondary outcome was the EQ-5D, a standardised measure of health-related quality of life. [22] The EQ-5D-5L descriptive system comprises five dimensions (mobility, self-care, usual activities, pain / discomfort, and anxiety / depression). For each dimension, there are 5 possible responses (no problems, slight problems, moderate problems, severe problems, unable to/extreme problems). The responses are coded to give a 5-digit code to describe the respondent's health state (such as 13254). Preference weights from the UK general population are applied to the resulting health states to produce a single summary index score for health status. EQ-5D-5L score is a measure anchored at 0 (representing 'death') and 1 ('full health'). This measure can include negative values, which reflect health states judged worse than death.

Explanatory Variables

Patient Demographics

The data collected in the Patient Demographic Questionnaire included patient reported age, gender, ethnicity, highest level of education and postcode. Patient age and gender were also reported by the clinic when registering the patient to use the DHI. Early versions of the DHI did not include the demographic questionnaire, which became available to all patients in April 2021. Where both clinic and patient-reported data were available, patient-reported age, gender and ethnicity were used, with clinic-reported data used as back-up.

For peer review only - http://bmjopen.bmj.com/site/about/guidelines.xhtml

To keep the data pseudo-anonymised, the Index of Multiple Deprivation (IMD) was provided to the study statistician, rather than the patient postcode. The English Indices of Deprivation (2019) was used to provide the Index of Multiple Deprivation (IMD) from the patient's postcode.[23] The IMD decile was not provided for 35 patients who had completed the demographic questionnaire. These were either entered incorrectly or were new, so not in the latest update of the IMD registry. Additionally, patient date of birth (as supplied by the clinic) was replaced with year of birth, from which an approximate age could be calculated.

Patient Reported Outcome Measures (PROMs)

In this study, six validated questionnaires were used to capture the severity of five of the core symptoms of PCS through patient-reported outcome measures (PROMs). The PROMs were completed by patients based on their clinical need, as determined by the patient themselves or with their health care professional. The first PROM completed by the patient was taken as their baseline measurement. The date and time of completion in relation to when the patient first registered to use the DHI was recorded, along with the outcome scores. PROMs were analysed as continuous variables, unless stated otherwise. Where threshold values for caseness are available, we present the number of patients within each of these categories to enable comparison between this study and other research.

1. Breathlessness
a) Dyspnoea-12 gives an overall score of breathlessness impact, with higher scores corresponding to greater severity. [24-26]
b) MRC Dyspnoea Scale measures the degree of breathlessness related to activity, with higher scores corresponding to greater severity. [27 28] The scale takes the values 1 to 5, using the following classifications: MRC 1 (Mild); MRC 2 to 3 (Moderate) and MRC 4 to 5 (Severe).[29] We analysed this variable as a categorical score.

2. Fatigue
Functional Assessment of Chronic Illness Therapy – Fatigue (FACIT-F) measures self-reported fatigue and its impact on daily activities and function with lower scores corresponding to greater fatigue. A threshold value of 30 was chosen in line with fatigue reported in a cancer population. [26] Population mean value for FACIT-F in the general population has been reported as 43. [25 26 30]

3. Anxiety
The Generalized Anxiety Disorder scale (GAD-7) is used as a screening tool and severity measure for anxiety. [31] A cut off value of 10 or more identifies anxiety. Additionally, threshold values are also considered: No anxiety (0-4); Mild anxiety (5 to 9); Moderate anxiety (10 to 14) and Severe anxiety (15 to 21).

4. Cognition (brain fog)
The Perceived Deficits Questionnaire, 5 item version (PDQ-5) measures the degree to which individuals perceive themselves as experiencing cognitive difficulties [32 33]. Higher scores

For peer review only - http://bmjopen.bmj.com/site/about/guidelines.xhtml

indicate more perceived deficits. The following threshold values suggested by Lam[34] are used: Minimal 0-8; Moderate 9-14; Severe 15-20.

5. Depression
The Patient Health Questionnaire eight item depression scale (PHQ-8) was chosen over the 9-item (PHQ-9) PROM for this study as it was not always certain that adequate intervention would be available if the question on suicidal thoughts or self-harm was endorsed; therefore, this question was omitted. [35] The same scoring thresholds are used as for PHQ-9, with a score of 10 or more used as a cut off for a diagnosis of depression. [36]

Statistical Analysis

Primary Outcome

Logistic regression was used to identify the PROMs associated with a high WSAS score (≥20) after accounting for the effects of demographic variables. First, we built a model for the demographic factors associated with high WSAS score. Age and gender were included as covariates in all models. Other demographics, including highest level of education, ethnicity (as white or non-white) and IMD quintile, were added using a stepwise approach based on the Likelihood Ratio (LR) Test. Any demographic variables with a p-value below 0.2 were retained for inclusion in subsequent models. At each stage, the McKelvey and Zavoina's R-squared value of the model including the additional term was calculated as a measure of the proportion of variation in the binary WSAS outcome attributable to the selected factors[37].

The FACIT-F score was reversed (calculated as 52 minus reported score), to align the direction of the score with other variables in the analysis. Higher values of the score now represent greater fatigue. We refer to this as FACIT-F (reversed scale).

Next, we added each of the PROMs (Dyspnoea-12, MRC-Dyspnoea, FACIT-F (reversed scale), GAD-7, PDQ-5, and PHQ-8) in a univariable fashion to the logistic regression model for the demographic factors. Any PROMs with a p-value below 0.2 were retained for potential inclusion in subsequent models. A multivariable model including both demographics and PROMs was developed by sequentially adding or removing PROMs according to the LR test using a p-value threshold of 0.05. The McKelvey and Zavoina's R-squared value was calculated at each stage as a measure of model fit. For the final model, we calculated the reduction in R-squared from removing each PROM from the model as a measure of the contribution of that variable to explaining variance in the WSAS outcome. Standardised effect estimates were produced to facilitate comparisons between the effect sizes of the PROMs, as they were each measured on different scales.

The analysis was conducted using a complete cases approach, assuming data were missing at random (MAR) conditional on the variables included in the regression models. Comparisons were made between the demographic characteristics of the full sample of treatment-seeking patients and those providing a baseline WSAS measure to assess the potential for selection bias due to the exclusion of patients with missing WSAS scores.

For peer review only - http://bmjopen.bmj.com/site/about/guidelines.xhtml

Secondary Outcomes

WSAS Domain score analysis

Secondary analysis was conducted to assess the extent to which the PROMs identified in the main analysis were associated with the individual domain scores of each of the 5 WSAS domains. The PROMs used in the multivariable logistic model were tested as explanatory variables in linear regression models for each of the 5 domains of ability to work, home management, social leisure activities, private leisure activities and close relationships. Models were adjusted for age and gender as in the primary analysis. Standardised estimates of effect size and change in adjusted R-squared values were calculated for each PROM in the multivariable model.

EQ-5D-5L analysis

Frequencies and proportions of patients reporting each dimension and level of EQ-5D-5L were calculated. Linear regression analysis of the EQ-5D index score was carried out to quantify the effect of patient demographics and PROMs on health-related quality of life (HRQoL). Multivariable linear regression models for the EQ-5D-5L analysis were developed adopting the same model selection strategy used in the primary analysis.

Working days lost due to Post-COVID syndrome

Additionally, LWCR users were asked to complete a study-specific questionnaire to capture data on the number of working days lost in the 28 days prior to questionnaire completion. Users were asked "In the last 4 weeks how many days off work (sick leave) have you taken due to Covid-19 and/or rehabilitation." The correlation between the number of working days lost and the WSAS 'work' domain was estimated.

All analyses were carried out in Stata version 17.0.

Patient and Public Involvement

This study had substantial PPI involvement with co-investigator (JB), steering group (JB, KB), individual work package management groups and an overall PPI Advisory Group. The feedback from PPI at an early stage was essential in determining the PROMs chosen in the study and the primary outcome measure of the WSAS. [20]

RESULTS

Patient Demographics

The study included 3754 treatment-seeking PCS patients with a mean age of 47.7 (SD 12.3) years, and 3541 (94.4%) being of working age (18–65) from across 31 clinics in the UK. The population were 71% (n=2675) female and 87% (n =2414) of White ethnicity (Table 1) and skewed toward affluence, with 11% (n =289) from the most deprived quintile and 24% (n=642) from the least deprived. Just over a half (n=1466, 53%) were educated to degree level or higher. Similar patient characteristics were seen in those who completed the WSAS and EQ-5D PROMs compared to the overall sample of patients using the app (Table 1).

Table 1: Sociodemographic characteristics of the patients in the study

For peer review only - http://bmjopen.bmj.com/site/about/guidelines.xhtml

Patient characteristic<br>n (%) unless stated otherwise	Study population<br>n (%)<br>(N=3754)	WSAS completed<br>n (%)<br>(n=2627)	EQ-5D-5L completed<br>n (%)<br>(n=2643)
Age (years), mean (SD)	47.7 (12.3)<br>(n=3753)	47.2 (11.9)	47.2 (11.9)
Age category (years)			
18 – 29	349 (9.3)	236 (9.0)	237 (9.0)
30 – 39	615 (16.4)	439 (16.7)	440 (16.6)
40 – 49	1084 (28.9)	771 (29.3)	773 (29.2)
50 – 59	1127 (30.0)	815 (31.0)	820 (31.0)
60 – 69	469 (12.5)	310 (11.8)	317 (12.0)
70 and over	109 (2.9)	56 (2.1)	56 (2.1)
Missing*	1	0	0
Gender			
Female	2675 (71.3)	1898 (72.3)	1909 (72.3)
Male	1060 (28.2)	719 (27.4)	724 (27.4)
Non-binary	10 (0.3)	9 (0.3)	9 (0.3)
Missing*	9	1	1
Highest Educational Level			
No education	113 (4.1)	106 (4.1)	102 (4.0)
School leaver (NVQ 1-2)	611 (22.1)	574 (22.5)	574 (22.6)
A-Level (NVQ-3)	574 (20.8)	532 (20.8)	533 (21.0)
Degree (NVQ-4)	581 (21.0)	527 (20.6)	526 (20.7)
Postgraduate Degree (NVQ-5)	885 (32.0)	817 (32.0)	808 (31.8)
Missing*	990	71	100
Ethnicity			
White	2414 (87.3)	2242 (87.7)	2234 (87.8)
Asian or Asian British	177 (6.4)	159 (6.2)	155 (6.1)

For peer review only - http://bmjopen.bmj.com/site/about/guidelines.xhtml

Black African Caribbean or Black British	55 (2.0)	48 (1.9)	47 (1.8)
Mixed or Multiple Ethnicity	67 (2.4)	61 (2.4)	62 (2.4)
Other ethnic group	32 (1.2)	27 (1.1)	26 (1.0)
Prefer not to say	19 (0.7)	19 (0.7)	19 (.7)
Missing*	990	71	100
IMD Quintile			
1 to 2 (20 % most deprived)	289 (10.6)	274 (10.9)	272 (10.8)
3 to 4	537 (19.7)	500 (19.8)	491 (19.6)
5 to 6	657 (24.1)	610 (24.2)	606 (24.1)
7 to 8	604 (22.1)	555 (22.0)	556 (22.1)
9 to 10 (20% least deprived)	642 (23.5)	585 (23.2)	586 (23.3)
Missing*	1025	103	132

* Data on patient-reported characteristics is missing for 990 who did not complete the Patient Demographics questionnaire. In addition, a further 35 are missing IMD as their IMD decile was not available. Percentages do not include those with missing values in the denominator

The functional impairment and quality of life of the treatment seeking PCS population

Functional impairment

Characteristics of patients who completed the WSAS PROM were similar to those of all users of the LWCR DHI (Table 1). The population reported a very high degree of functional impairment (mean WSAS score of 20.6, n=2627), with over half the patients (53%) scoring above 20 in the moderately severe category (Appendix 1, Appendix Figure 1). Functional impairment was seen across all five of the WSAS domains; with the highest rates of functional impairment seen in the Social Leisure Activities and Ability to Work categories; mean scores 4.7 and 4.6, respectively. The least affected domain in PCS patients was close relationships with a mean score of 3.0 (Appendix 1).

For peer review only - http://bmjopen.bmj.com/site/about/guidelines.xhtml

For peer review only

Health related quality of life

EQ-5D data was completed by 2643 LWCR DHI users. Patients reported a large impact on health-related quality of life, with an average (median) EQ-5D index score of 0.60 (IQR 0.41 to 0.71) (Appendix Figure 2).

Appendix 2 shows the number of respondents reporting a problem in each domain. The two domains of the EQ-5D most affected by PCS were pain/discomfort reported by 2542 (96.2%) and anxiety/depression reported by 2509 (95%). The least affected EQ-5D domain was usual activities, with 36% reporting no problems.

Working days lost due to Post-COVID syndrome

Half (n=1321/2600, 50.8%) of patients who completed the study-specific questionnaire reported losing one or more days from work in the previous month, with a fifth (20.3%) reporting between 20 and 28 working days lost. (Appendix 3) Correlation between the baseline WSAS work domain (score 0 to 8) and number of working days lost was 0.52, showing moderate correlation.

Severity of patient reported symptoms

The LWCR DHI users were extremely fatigued, reporting a mean FACIT-F score of 19.6, well below the threshold value of 30 used in this study. (FACIT-F reversed scale mean 32.4; threshold value of 22). Mental health was affected, with a mean GAD-7 score of 9 (corresponding to mild anxiety) and a mean PHQ-8 of 11.8, meeting the clinical threshold for depression. Additionally, breathlessness was evident, with a mean Dyspnoea-12 score of 12 and median (IQR) MRC Dyspnoea Scale score of 2 (2,3). The PCS population also reported moderate cognitive difficulties (brain fog) with a mean PDQ-5 score of 12. (Table 2).

For peer review only - http://bmjopen.bmj.com/site/about/guidelines.xhtml

Table 2: Summary of Patient Reported Outcome Measures (PROMs) and scores for users of the Living With Covid Recovery DHI. Summary measures of overall mean (SD) and number (%) within each threshold category are reported.

PROM	Measures	Number completed	Mean (SD)	Threshold values<br>[Number in each threshold category (%)]
Work and Social Adjustment Score (WSAS)<br>***Primary Outcome***	Functional limitations of the patient. Higher scores indicate greater functional impairment.<br>Range:0-40	2627	20.6 (9.9)	<10: subclinical [394 (15.0)]<br>10 – 19: significant [843 (32.1)]<br>>20: Moderately severe [1390 (52.9)]
Ability to work*	Functional limitations within domains.<br>Subscale range: 0-8<br>0: not at all affected to<br>8: very severely affected	2621	4.6 (2.4)	
Home management		2627	4.2 (2.2)	
Social leisure activities		2627	4.0 (2.2)	
Private leisure activities		2627	4.7 (2.3)	
Close relationships		2627	3.0 (2.4)	
EQ-5D (EQ-5D-5L)<br>***Secondary Outcome***	A standardised measure of health status	2633	0.54 (0.27)	
**Explanatory variables**				
Functional Assessment of Chronic Illness Therapy – Fatigue (FACIT-F)	Self-reported fatigue and its impact upon daily activities and function. Higher scores indicate less fatigue.	2890	19.6 (10.1)	<30: Impairment [2418 (83.7)]<br>≥30: No impairment [472 (16.3)]

For peer review only - http://bmjopen.bmj.com/site/about/guidelines.xhtml

	Range: 0-52			
FACIT-F (reversed scale)<br>Scale reversed in results to aid interpretation	Higher scores indicate greater fatigue.<br>Range: 0-52.	2890	32.4 (10.1)	≤22: No impairment [472 (16.3)]<br>>22: Impairment [2418 (83.7)]
Generalized Anxiety Disorder scale<br>(GAD-7)	Screening tool and severity measure for anxiety.<br>Range: 0-21	2774	9.0 (5.9)	<4: No anxiety [715 (25.8)]<br>5-9: Mild anxiety [870 (31.4)]<br>10-14: Moderate anxiety [591 (21.3)]<br>≥15: Severe anxiety [598 (21.6)]
Patient Health Questionnaire depression scale (PHQ-8)	A valid diagnostic and severity measure for current depressive disorders. Higher scores indicate more severe depression.<br>Range: 0-24	2661	11.8 (6.0)	<10: No depression [1034 (38.9)]<br>≥10: Clinical depression [1627 (61.1)]
Dyspnoea-12	Overall score of breathlessness impact, with higher scores corresponding to greater severity.<br>Range: 0 to 36	2656	12.0 (9.3)	No threshold values
MRC Dyspnoea Scale (Median (IQR))	Degree of breathlessness related to activity, with higher scores corresponding to greater severity.<br>Range: 1 to 5	2607	2 (2,3)	1: Mild [262 (10.1)]<br>2-3: Moderate [1800 (69.0)]<br>4-5: Severe [545 (20.9)]
Perceived Deficits Questionnaire, 5 item version (PDQ-5)	Measures the degree to which individuals perceive themselves as experiencing cognitive difficulties. Higher scores indicate more perceived deficits.	2783	12.3 (4.3)	≤8: Minimal [519 (18.7)]<br>9-14: Moderate [1346 (48.4)]<br>≥15: Severe [918 (33.0)]

For peer review only - http://bmjopen.bmj.com/site/about/guidelines.xhtml

	Range: 0-20			

* Reduced number of completed answers as patients who had retired or chose not to work did not need to answer this question.

For peer review only

For peer review only - http://bmjopen.bmj.com/site/about/guidelines.xhtml

Contribution of Fatigue to functional impairment and health related quality of life

Functional impairment

Fatigue, depression, and cognitive impairment were significant predictors of a high WSAS (functional impairment) score. Fatigue was the strongest predictor of high WSAS, with a one-point increase in the reversed FACIT-F associated with an increase of 16% in the odds of a patient having a high WSAS score. When sequentially removing each PROM from the final multivariable model, the greatest contribution to reduction in R-squared (measure of goodness of fit of the statistical model) was attained by the removal of FACIT-F (33.8%), compared to a 1.7% reduction in R-squared for both PHQ-8 and PDQ-5) (Table 3).

Figure 1a shows the heat map distribution of WSAS scores with almost all the high scores (denoted by pink squares) above the FACIT-F threshold for impairment. In contrast, the high WSAS scores are spread more evenly across both sides of the cognition and depression threshold of 10 for PDQ-5 and PHQ-8 respectively (Figures 1a and 1b). FACIT-F also contributed strongly to the scores for each of the five WSAS domains, with PHQ-8 only making a substantive contribution, outperforming that of FACIT-F, in the ‘close relationships’ domain. The contribution of PDQ-5 was small compared to FACIT-F, with ability to work most associated with cognition. (Figure 2).

There was no significant difference in the functional impairment between genders, but a higher rate of functional impairment was seen in the younger age groups. The highest rate was seen in the 30-39 age group, compared to the reference age category of age 18 to 29 **(**OR 1.18, 95% CI 0.78 to 1.77; **Table 3)**.

For peer review only

Table 3: WSAS multivariable model for different patient characteristics and PROM scores (N=2556)

**Patient Characteristics**		**Odds Ratio** (95% CI)	**p-value**	**Reduction in R-squared** [Full model R^2= 0.529]	**Standardised effect size**
**Age**	18 – 29	Reference			
	30 – 39	1.18 (0.78, 1.77)	0.441		
	40 – 49	0.90 (0.62, 1.32)	0.603		
	50 – 59	0.62 (0.42, 0.90)	0.011		
	60 – 69	0.55 (0.35, 0.85)	0.008		
	70 and over	0.26 (0.12, 0.59)	0.001		
**Gender**	Male	Reference			
	Female	0.83 (0.66,1.05)	0.115		
	Non-binary	0.25 (0.05, 1.17)	0.078		
**PROMs**	FACIT-F (reversed scale) High values indicate greater fatigue	1.16 (1.14, 1.18)	<0.001	0.179	4.47
	PHQ-8 High values indicate more severe depression	1.05 (1.03, 1.08)	<0.001	0.009	1.37
	PDQ-5	1.06 (1.03, 1.09)	<0.001	0.009	1.29

For peer review only - http://bmjopen.bmj.com/site/about/guidelines.xhtml

	High values indicate more perceived deficits				

For peer review only

Health related quality of life

Fatigue also contributed to the health-related quality of life of PCS patients with the FACIT-F (reversed scale) being a significant predictor of the EQ-5D index score. FACIT-F (reversed scale) made the largest contribution to explaining variation in quality of life (change in R-squared of 8.4% compared to 5.6% for MRC Dyspnoea Scale, 3.1% for GAD-7, 1.7% for PHQ-8 and 0.5% for Dyspnoea-12. (Appendix 1).

DISCUSSION

Principal findings

Treatment seeking Post-COVID patients consisting of mainly female, white, working age, and well-educated people are experiencing striking levels of functional impairment and low health-related quality of life. This impairment is mainly driven by their fatigue level, causing significant impact on their ability to work and care for others.

The patients report levels of functional impairment worse than in several other known clinical cohorts, such as patients referred to IAPT services in the South West of the UK (mean score 18.8 at referral). [38] Functional impairment was worse than in stroke patients (mean WSAS scores of 16) and comparable to patients with Parkinson's Disease (the mean WSAS scores ranged from 22.9 to 24.8), both debilitating neurological conditions. [39] Similarly, these patients report low Health-Related Quality of Life (HRQoL), with a mean EQ-5D score of 0.54 (SD 0.26), which compares poorly with patients with advanced/metastatic cancers.[40 41]For example, mean EQ-5D for stage IV lung cancer was between 0.66 and 0.84.[41] The results of the multivariable analysis show that fatigue is the strongest predictor of functional impairment (Table 3) and health-related quality of life (Appendix 4). Our population of patients reported worse fatigue (mean score of FACIT-F 19.6) than patients with stroke (mean score 38), inflammatory bowel disease (mean score 38.9), end stage renal disease (mean score 39) and even anaemic cancer patients (mean score 24)[30 42-45] As well as patients reporting severe fatigue, they also report breathlessness, anxiety, depression and cognitive dysfunction.

This study is, to the best of our knowledge, the first reporting on functional limitations and health related quality of life in PCS from a national population of patients referred for specialist rehabilitation. As such, they differ from other cohort studies, which have followed up patients initially identified as hospitalised acute COVID patients (mean FACIT-F score 16.8) or through positive COVID testing in the general public.[46] One study has recently reported on a single centre Post-COVID assessment clinic showing similar levels of fatigue, but using a different measure (mean Fatigue Assessment Scale score 29) and inability of patients to work across 19hospitalised and non-hospitalised patients [47]. None of the other studies have reported on functional impairment using the WSAS which measures the impact PCS is having on patients' normal daily activities.

This study enforces the recommendation for the use of a consistent set of outcome measures in studies in COVID-19. One such list of recommended variables is the ICHOM Set of Patient-Centered Outcome Measures for COVID-19 which recommends that research assesses functional status, quality of life and social functioning in addition to the typically

For peer review only

reported measures of clinical outcomes, mental functioning, and symptom reporting. [48 49] Additionally, consideration should be given to the interpretation of fatigue in PCS patients, as advised by Sandler et al. [10] Patients may report fatigue when experiencing weakness, dyspnoea, cognitive dysfunction, somnolence or low mood.

Strengths and limitations of this study

All the data collected in this study were recorded in real time by patients and used by clinicians in their assessment and treatment. All PROMs used in the LWCR study were validated measures selected to provide the most reliable clinical information for patient benefit. Using these outcome measures allowed patient scores to be compared across disease types and with scores from other COVID studies. This necessity for clinically led data collection led to substantial missing data, partly due to the DHI evolving to include new features over the reported period; patients who used the DHI later in its development were able to complete more PROMs. The primary reason for App usage and associated data collection was not for research – as a result data on the severity of the initial disease or COVID-19 vaccination status were not collected within the app. Other studies have reported on the inconsistent relationship between severity of initial disease and severity of PCS, [46 50] therefore we did not seek to capture further patient data from other sources.

Our chosen approach to the regression analysis was to use the observed data (a complete cases approach) but we acknowledge that exclusion of the missing data may have introduced bias. An alternative approach to analysing data that are missing at random would be to use multiple imputation but it has been recommended that complete cases analysis can be used as the primary analysis in situations where missing data is restricted to the dependent variable (we found very low levels of missing data in the explanatory variables when excluding patients with missing outcome data) and auxiliary variables have not been identified. [51]

Patients recruited to this study were sampled from the 31 specialist Post-COVID clinics that had chosen to use the LWCR DHI at the time of data extraction. Our sample is representative of the patients who are seen in PCS clinics nationally. The data may not be representative of all patients with Long COVID or PCS as many of these patients are not seen in a PCS clinic for a variety of reasons. This can be noted in the patient demographics which shows that the majority of our patients are white, affluent, and well-educated people. These patients are more likely to seek, and obtain, help than their counterparts.

This study has implications for the targeting of limited resources to effectively address functional limitations from PCS. Of particular concern is the large proportion of working age women in our study population, people who contribute substantially to the health care, social care and informal care sectors[52] at a time when these sectors are already under duress.[53] Post-COVID syndrome is clearly a multifactorial disease affecting physical and mental wellbeing but Post-COVID assessment services should consider focusing on assessing and treating fatigue to maximise the recovery and return to work in this large cohort of patients. Further work is needed to explore the recovery trajectories of this cohort over time and whether fatigue continues to predict functional impairment and low health-related quality of life over time.

For peer review only

CONCLUSION

In this first UK national study reporting clinical symptoms from patients referred for assessment and treatment of Post-COVID syndrome, we demonstrate high levels of functional impairment and low health-related quality of life. Fatigue appears to be the symptom most strongly associated with functional impairment. Currently, clinical services lack evidence-based approaches in treating patients experiencing fatigue related to PCS with no standard rehabilitation pathway. [11-14] This requires further targeted research. Our future work to explore the recovery trajectory of patients using the LWCR DHI may help to establish the extent to which WSAS, and other PROMs are sensitive to changes in the health of a patient with PCS. This work can contribute to the identification of PROMs best suited for use in assessing, managing, and treating patients with PCS, both digitally and in face-to-face appointments.

Author statement

EM and HG were responsible for the concept of the Living With Covid Recovery study. HG is the guarantor. SW was the first author of the manuscript and revised it after review from the wider study team. SW, WH, HG and MG advised on appropriate statistical design. SW and WH carried out the statistical analysis for the study. PP supported SW in preparing the paper for publication, including performing the literature search and drafting parts of the manuscript. All authors contributed to study design, reporting and review of the paper in Steering Committee meetings and reviewed the paper prior to submission.

Funding Statement

This study is funded by the National Institute for Health and Care Research (NIHR) Cross-programme [HS&DR] COVID-19 [project reference NIHR132243]. The views expressed are those of the authors and not necessarily those of the NIHR or the Department of Health and Social Care. This report is independent research supported by the NIHR ARC North Thames, NIHR ARC Wessex and NIHR ARC West.

For the purpose of open access, the author has applied a Creative Commons Attribution (CC BY) licence to any Author Accepted Manuscript version arising.

Competing interests

JB reports payments from University College London (UCL) for working as a PPI to prepare content for the DHI since May 2020. KB's research portfolio is part funded by NIHR Applied Research Collaboration Wessex. HG reports working as a Clinical Safety Officer for *Living With*. JRH reports receiving personal fees and fees to institution for honorariums and consultancy payments from AstraZeneca, Boehringer Ingelheim, Chiesi, GlaxoSmithKline, Takeda. JRH also reports receiving sponsorship for attending meetings from AstraZeneca and GlaxoSmithKline. HH reports payment from University of East London for providing a lecture on Long COVID and COVID Recovery in February 2021. SL reports grants from NIHR in which the payment was made to Camden and Islington NHS Trust between the period of October to September 2022. PEP reports grants from the Medical Research Council (MRC) and NIHR outside the submitted work. All other authors declare no competing interests.

For peer review only

Transparency declaration: Sarah Walker affirms that the manuscript is an honest, accurate, and transparent account of the study being reported; that no important aspects of the study have been omitted; and that any discrepancies from the study as planned have been explained.

Data sharing: To request access to the underlying research data, please contact Dr Henry Goodfellow henry.goodfellow.12@ucl.ac.uk.

Ethics

Ethical approval obtained from East Midlands – Derby Research Ethics Committee (reference 288199).

For peer review only

REFERENCES

1. National Institute for Health and Care Excellence. COVID-19 rapid guideline: managing the long-term effects of COVID-19 2022 [updated March 03. Available from: https://www.nice.org.uk/guidance/ng188.
2. World Health Organization. WHO Coronavirus (COVID-19) Dashboard 2022 [updated 2 Aug 2022; cited 2022 Aug 2]. Available from: https://covid19.who.int/ accessed 2 Aug 2022.
3. Maxwell E, Poole R. NIHR Themed Review: Living with Covid19 - Second review: National Institute of Health and Care Research; 2021 [updated 16 March. Available from: https://evidence.nihr.ac.uk/themedreview/living-with-covid19-second-review/ accessed 14 April 2022.
4. Whitaker M, Elliott J, Chadeau-Hyam M, et al. Persistent symptoms following SARS-CoV-2 infection in a random community sample of 508,707 people *medRxiv 21259452 [Preprint]* July 03, 2021 [cited 2022 Apr 14] doi: https://doi.org/10.1101/2021.06.28.21259452
5. Thompson EJ, Williams DM, Walker AJ, et al. Long COVID burden and risk factors in 10 UK longitudinal studies and electronic health records. *Nature Communications* 2022;13(1):3528. doi: 10.1038/s41467-022-30836-0
6. Office for National Statistics. Prevalence of ongoing symptoms following coronavirus (COVID-19) infection in the UK: 7 July 2022 [Available from: https://www.ons.gov.uk/peoplepopulationandcommunity/healthandsocialcare/conditionsanddiseases/bulletins/prevalenceofongoingsymptomsfollowingcoronaviruscovid19infectionintheuk/7july2022 accessed 18 July 2022.
7. Davis HE, Assaf GS, McCorkell L, et al. Characterizing long COVID in an international cohort: 7 months of symptoms and their impact. *EClinicalMedicine* 2021;38:101019. doi: 10.1016/j.eclinm.2021.101019 [published Online First: 20210715]
8. Boscolo-Rizzo P, Guida F, Polesel J, et al. Sequelae in adults at 12 months after mild-to-moderatecoronavirus disease 2019 (COVID-19). *Int Forum Allergy Rhinol* 2021;11:1685-88. doi: 10.1101/2021.04.12.21255343
9. Thompson EJ, Williams DM, Walker AJ, et al. Risk factors for Long-COVID : analyses of 10 longitudinal studies and electronic health records in the UK. *medRxiv 21259277 [Preprint]* July 10, 2021 [cited Nov 21, 2021] doi: 10.1101/2021.06.24.21259277
10. Sandler CX, Wyller VBB, Moss-Morris R, et al. Long COVID and Post-infective Fatigue Syndrome: A Review. *Open Forum Infectious Diseases* 2021;8(10) doi: 10.1093/ofid/ofab440
11. Sivan M, Greenhalgh T, Milne R, et al. Are vaccines a potential treatment for long covid? *BMJ* 2022;377:o988. doi: 10.1136/bmj.o988
12. Torjesen I. Covid-19 patients discharged from hospital have "substantially higher risk" of adverse outcomes and need monitoring. *BMJ* 2022;376:o265. doi: 10.1136/bmj.o265
13. Wise J. Covid-19: Long covid risk is lower with omicron than delta, researchers find. *BMJ* 2022;377:o1500. doi: 10.1136/bmj.o1500
14. Zimmermann P, Pittet LF, Curtis N. Long covid in children and adolescents. *BMJ* 2022;376:o143. doi: 10.1136/bmj.o143
15. Jones R, Davis A, Stanley B, et al. Risk Predictors and Symptom Features of Long COVID Within a Broad Primary Care Patient Population Including Both Tested and Untested Patients. *Pragmat Obs Res* 2021;12:93-104. doi: 10.2147/POR.S316186 [published Online First: 20210811]
16. Sudre CH, Murray B, Varsavsky T, et al. Attributes and predictors of long COVID. *Nat Med* 2021;27(4):626-31. doi: 10.1038/s41591-021-01292-y [published Online First: 2021/03/12]
17. Waters T, Wernham T. Long COVID and the labour market: The Institute for Fiscal Studies, 2022.
18. NHS England and NHS Improvement. Long-COVID: the NHS plan for 2021/22 2021 [updated June. Available from: https://www.england.nhs.uk/coronavirus/wp-content/uploads/sites/52/2021/06/C1312-long-covid-plan-june-2021.pdf accessed 21 November 2022.

For peer review only

19. Thorlby R, Gardner T, Allen L, et al. The NHS Long Term Plan and COVID-19: Assessing progress and the pandemic's impact. London: The Health Foundation, 2021.
20. Murray E, Goodfellow H, Bindman J, et al. Development, deployment and evaluation of digitally enabled, remote, supported rehabilitation for people with long COVID-19 (Living With COVID-19 Recovery): protocol for a mixed-methods study. *BMJ Open* 2022;12(2):1-9. doi: 10.1136/bmjopen-2021-057408 [published Online First: 20220207]
21. Mundt JC MA, Shear MK, Greist JH. The Work and Social Adjustment Scale : a simple measure of impairment in functioning. *Br J Psychiatry* 2002;180(46):61-4. doi: 10.1192/bjp.180.5.461
22. EuroQol Research Foundation. EQ-5D-5L Userguide, 2019.
23. Ministry of Housing CaLG. English indices of deprivation 2019: Research report, 2019.
24. Yorke J, Moosavi SH, Shuldham C, et al. Quantification of dyspnoea using descriptors: development and initial testing of the Dyspnoea-12. *Thorax* 2010;65(1):21-6. doi: 10.1136/thx.2009.118521 [published Online First: 20091208]
25. Montan I, Löwe B, Cella D, et al. General Population Norms for the Functional Assessment of Chronic Illness Therapy (FACIT)-Fatigue Scale. *Value Health* 2018;21(11):1313-21. doi: 10.1016/j.jval.2018.03.013 [published Online First: 2018/11/18]
26. Piper BF, Cella D. Cancer-related fatigue: Definitions and clinical subtypes. *J Natl Compr Cancer Netw* 2010;8(8):958-66. doi: 10.6004/jnccn.2010.0070
27. Bestall JC PE, Garrod R, Garnham R, Jones PW, Wedzicha JA. Usefulness of the Medical Research Council (MRC) dyspnoea scale as a measure of disability in patients with chronic obstructive pulmonary disease. *Thorax* 1999;54:581-6. doi: 10.1136/thx.54.7.581
28. Horwitz LI, Garry K, Prete AM, et al. Six-Month Outcomes in Patients Hospitalized with Severe COVID-19. *J Gen Intern Med* 2021;36(12):3772-77. doi: 10.1007/s11606-021-07032-9 [published Online First: 20210805]
29. Paladini L, Hodder R, Cecchini I, et al. The MRC dyspnoea scale by telephone interview to monitor health status in elderly COPD patients. *Respir Med* 2010;104(7):1027-34. doi: 10.1016/j.rmed.2009.12.012
30. Cella D LJ, Chang CH, Peterman A, Slavin M. Fatigue in cancer patients compared with fatigue in the general United States population. *Cancer* 2002;94:528-38. doi: https://doi.org/10.1002/cncr.10245
31. Spitzer RL, Kroenke K, Williams JBW, et al. A Brief Measure for Assessing Generalized Anxiety Disorder The GAD-7. *Arch Intern Med* 2006;166(10):1092-7. doi: 10.1001/archinte.166.10.1092
32. Sullivan M, Edgley K, DeHousx E. A survey of multiple sclerosis, part 1: perceived cognitive problems and compensatory strategy use. *Can J Rehabil* 1990;4(2):99-105.
33. Julian LJ, Yazdany J, Trupin L, et al. Validity of brief screening tools for cognitive impairment in rheumatoid arthritis and systemic lupus erythematosus. *Arthritis Care Res (Hoboken)* 2012;64(3):448-54. doi: 10.1002/acr.21566
34. Lam R. Subjective measures of cognitive dysfunction in major depressive disorder. In: McIntyre RS, ed. Cognitive Impairment in Major Depressive Disorder: Clinical Relevance, Biological Substrates, and Treatment Opportunities. Cambridge: Cambridge University Press 2016:242-50.
35. Kroenke K, Strine TW, Spitzer RL, et al. The PHQ-8 as a measure of current depression in the general population. *J Affect Disord* 2009;114(1-3):163-73. doi: 10.1016/j.jad.2008.06.026 [published Online First: 20080827]
36. Kroenke K, Spitzer R. The PHQ-9: a new depression and diagnostic severity measure. *Psychiatry* 2002;32(9):509-15. doi: https://doi.org/10.3928/0048-5713-20020901-06
37. McKelvey RD, Zavoina W. A statistical model for the analysis of ordinal level dependent variables. *J Math Sociol* 1975;4(1):103-20. doi: 10.1080/0022250X.1975.9989847

For peer review only

38. Zahra D, Qureshi A, Henley W, et al. The work and social adjustment scale: Reliability, sensitivity and value. *International Journal of Psychiatry in Clinical Practice* 2014;18(2):131-38. doi: 10.3109/13651501.2014.894072
39. Hommel M, Trabucco-Miguel, S, Joray, S, Naegele, B, Gonnet, N, Jaillard, A,. Social dysfunctioning after mild to moderate first-ever stroke at vocational age. *Journal of Neurology, Neurosurgery & Psychiatry* 2009;80(4):371-75. doi: 10.1136/jnnp.2008.157875.
40. Paracha N, Abdulla A, MacGilchrist KS. Systematic review of health state utility values in metastatic non-small cell lung cancer with a focus on previously treated patients. *Health Qual Life Outcomes* 2018;16(179) doi: 10.1186/s12955-018-0994-8
41. Pourrahmat MM, Kim A, Kansal AR, et al. Health state utility values by cancer stage: a systematic literature review. *Eur J Health Econ* 2021;22:1275-88. doi: 10.1007/s10198-021-01335-8
42. Acaster S, Dickerhoof R, DeBusk K, et al. Qualitative and quantitative validation of the FACIT-fatigue scale in iron deficiency anemia. *Health Qual Life Outcomes* 2015;13:60. doi: 10.1186/s12955-015-0257-x [published Online First: 2015/05/20]
43. Chen K, Marsh EB. Chronic post-stroke fatigue: It may no longer be about the stroke itself. *Clin Neurol Neurosurg* 2018;174:192-97. doi: https://doi.org/10.1016/j.clineuro.2018.09.027
44. Wang S-Y, Zang X-Y, Fu S-H, et al. Factors related to fatigue in Chinese patients with end-stage renal disease receiving maintenance hemodialysis: a multi-center cross-sectional study. *Ren Fail* 2015;38(3):442-50. doi: 10.3109/0886022X.2016.1138819
45. Christensen KR, Ainsworth MA, Steenholdt C, et al. Fatigue is a systemic extraintestinal disease manifestation largely independent of disease activity, chronicity, and nutritional deficiencies in inflammatory bowel disease on biologics. *Scand J Gastroenterol* 2022;57(9):1051-57. doi: 10.1080/00365521.2022.2060049
46. Evans RA, McAuley H, Harrison EM, et al. PHOSP-COVID Collaborative Group. Physical, cognitive, and mental health impacts of COVID-19 after hospitalisation (PHOSP-COVID): a UK multicentre, prospective cohort study. *Lancet Respir Med* 2021;9(11):1275-87. doi: 10.1016/S2213-2600(21)00383-0 [published Online First: 2021 Oct 7]
47. Heightman M, Prashar J, Hillman TE, et al. Post-COVID-19 assessment in a specialist clinical service: a 12-month, single-centre, prospective study in 1325 individuals. *BMJ Open Respiratory Research* 2021;8:e001041. doi: https://doi.org/10.1136/bmjresp-2021-001041
48. International Consortium for Health Outcomes Measurement (ICHOM). Sets of Patient-Centered Outcome Measures [Available from: https://connect.ichom.org/patient-centered-outcome-measures/ accessed 5th July 2022.
49. Munblit D, Nicholson T, Akrami A, et al. A core outcome set for post-COVID-19 condition in adults for use in clinical practice and research: an international Delphi consensus study. *Lancet Respir Med* 2022;10(7):715-24. doi: 10.1016/S2213-2600(22)00169-2.
50. Taquet M, Dercon Q, Luciano S, et al. Incidence, co-occurrence, and evolution of long-COVID features: A 6-month retrospective cohort study of 273,618 survivors of COVID-19. *PLoS Med* 2021;18(9):1-22. doi: http://dx.doi.org/10.1371/journal.pmed.1003773 [published Online First: 28 September 2021]
51. Jakobsen JC, Gluud C, Wetterslev J, et al. When and how should multiple imputation be used for handling missing data in randomised clinical trials – a practical guide with flowcharts. *BMC Medical Research Methodology* 2017;17(1):162. doi: 10.1186/s12874-017-0442-1
52. Devine BF, Foley N, Ward M. Women and Economy: House of Commons Library, 2021.
53. The Department of Health and Social Care (DHSC) and Office for National Statistics (ONS). Direct and Indirect Health Impacts of COVID-19 in England: The Department of Health and Social Care (DHSC) and Office for National Statistics (ONS), 2021.

For peer review only

Figures Legends:

Figure 1a: Heat Map showing the distribution of each patient's (n=2502) WSAS scores (higher score representing an increase in functional limitations) compared to their corresponding fatigue levels FACIT-F (reversed scale) and depression (PHQ-8) levels. The dashed line represents the threshold values for significant fatigue on the x-axis and clinical depression on the y-axis.

Figure 1b: Heat Map showing the distribution of each patient's (n=2520) WSAS scores (higher score representing an increase in functional limitations) compared to their corresponding fatigue levels (FACIT-F (reversed scale) and brain fog (PDQ5) levels. The dashed line represents the threshold value for significant fatigue on the x-axis and moderate brain fog on the y-axis.

Figure 2: Change in proportion of variation in WSAS explained (R-squared) when PROMs were removed from the linear regression models for each WSAS domain.

For peer review only

Figure 1a:

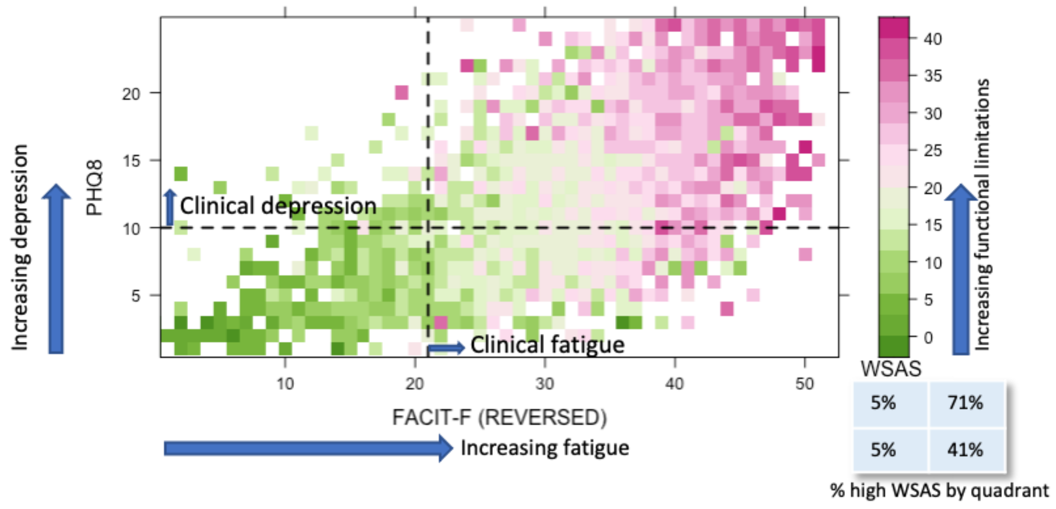

Figure 1b:

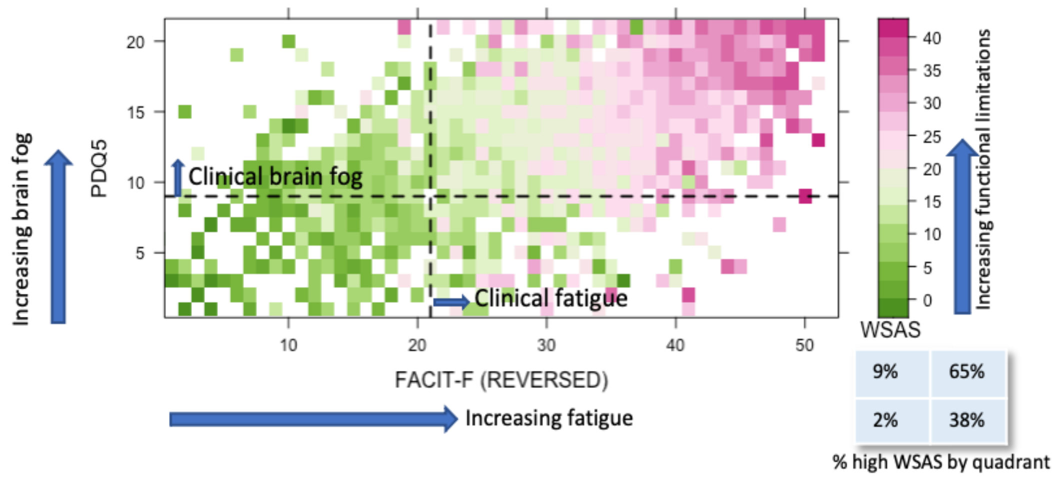

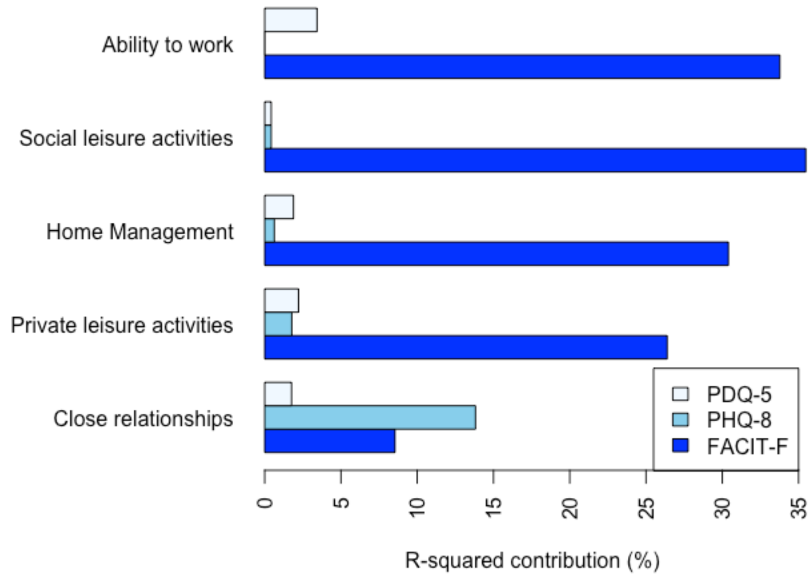
Ability to work
Social leisure activities
Home Management
Private leisure activities
Close relationships
PDQ-5
PHQ-8
FACIT-F
0
5
10
15
20
25
30
35
R-squared contribution (%)

Appendix

Appendix 1: Mean scores for the overall WSAS score and individual WSAS domains

WSAS Domain	N	Mean (SD)	Range and threshold values
WSAS overall score	2627	20.6 (9.9)	Range:0-40<br><10: subclinical<br>10 – 19: significant<br>**>20: Moderately severe**
Ability to work*	2621	4.6 (2.4)	Subscale range: 0-8<br>0: not at all affected to<br>8: very severely affected
Home management	2627	4.2 (2.2)	
Social leisure activities	2627	4.0 (2.2)	
Private leisure activities	2627	4.7 (2.3)	
Close relationships	2627	3.0 (2.4)	

* Reduced number of completed answers as patients who had retired or chose not to work did not need to answer this question.

For peer review only

Appendix 2: EQ-5D-5L frequencies and proportions reported by dimension and level

	Mobility n (%)	Self-care n (%)	Usual activities n (%)	Pain / discomfort n (%)	Anxiety / depression n (%)
**Level 1** (No problems)	712(26.9)	318(12.0)	959(36.3)	101(3.8)	134(5.1)
**Level 2** (Slight problems)	795(30.1)	1702(64.4)	250(9.5)	983(37.2)	701(26.5)
**Level 3** (Moderate problems)	309(11.7)	98(3.7)	506(19.1)	358(13.5)	675(25.5)
**Level 4** (Severe problems)	810(30.6)	511(19.3)	759(28.7)	373(14.1)	267(10.1)
**Level 5** (Extreme problems / unable to do)	17(0.6)	14(0.5)	169(6.4)	828(31.3)	866(32.8)
Total	2643(100)	2643(100)	2643(100)	2643(100)	2643(100)

For peer review only

Appendix 3: Working days lost due to Post-COVID syndrome in 28 days prior to completion of Service Use Questionnaire

Number completed Service Use questionnaire	2600
Number (%) who lost 1 or more days from work	1321 (50.8)
Mean number of working days lost (SD)*	13.8 (10.7)
Median number of working days lost (IQR)*	10 (4 to 28)

* in those who lost 1 or more days off work

Appendix 4: EQ-5D index score multivariable model for different patient characteristics and PROM scores (N=2405)

Patient Characteristics		Model coefficients (95% CI)	p-value	Change in R-squared * [Full model R-sq=0.573)	Standardised effect size
Age	18 to 29	Reference			
	30 to 39	-0.02 (-0.05, 0.01)	0.219		
	40 to 49	-0.03 (-0.06, -0.01)	0.009		
	50 to 59	-0.03 (-0.06, -0.01)	0.018		
	60 to 69	-0.06 (-0.09, -0.03)	<0.0001		
	70 and over	-0.07 (-0.12, -0.02)	0.005		
Gender	Male	Reference			
	Female	0.00 (-0.01, 0.02)	0.786		
Educational	No education	Reference			

For peer review only

level	School leaver (NVQ 1-2)	0.00 (-0.04, 0.04)	0.948		
	A-Level (NVQ-3)	0.02 (-0.02, 0.05)	0.389		
	Degree (NVQ-4)	0.01 (-0.02, 0.05)	0.464		
	Postgraduate degree (NVQ-5)	0.02 (-0.01, 0.06)	0.210		
Ethnicity	White	Reference			
	Non-white	-0.02 (-0.04, 0.00)	0.073		
IMD Quintile	1 (most deprived)	Reference			
	2	0.02 (0.00, 0.05)	0.059		
	3	0.03 (0.00, 0.05)	0.025		
	4	0.05 (0.02, 0.07)	<0.0001		
	5 (least deprived)	0.03 (0.01, 0.06)	0.008		
PROMs	FACIT-Fatigue (reversed scale)	-0.01 (-0.01, -0.01)	<0.0001	0.048	-0.080
	PHQ-8	-0.01 (-0.01, -0.01)	<0.0001	0.010	-0.044

For peer review only

	GAD-7	-0.01 (-0.01, -0.01)	<0.0001	0.018	-0.051
	MRC Dyspnoea Scale: Grade 1	Reference			
	MRC Dyspnoea Scale: Grade 2	0.02 (0.00, 0.04)	0.108		0.010
	MRC Dyspnoea Scale: Grade 3	-0.02 (-0.04, 0.01)	0.191	0.032	-0.009
	MRC Dyspnoea Scale: Grade 4	-0.08 (-0.11, -0.05)	<0.0001		-0.030
	MRC Dyspnoea Scale: Grade 5	-0.25 (-0.30, -0.20)	<0.0001		-0.045
	Dyspnoea-12	0.00 (0.00, 0.00)	<0.0001	0.003	-0.020

* Reduction in R-squared value when variable is removed from the final model. Overall model has R-squared value of 0.573

For peer review only

Appendix Figure 1: Frequency distribution of the first reported (baseline) WSAS

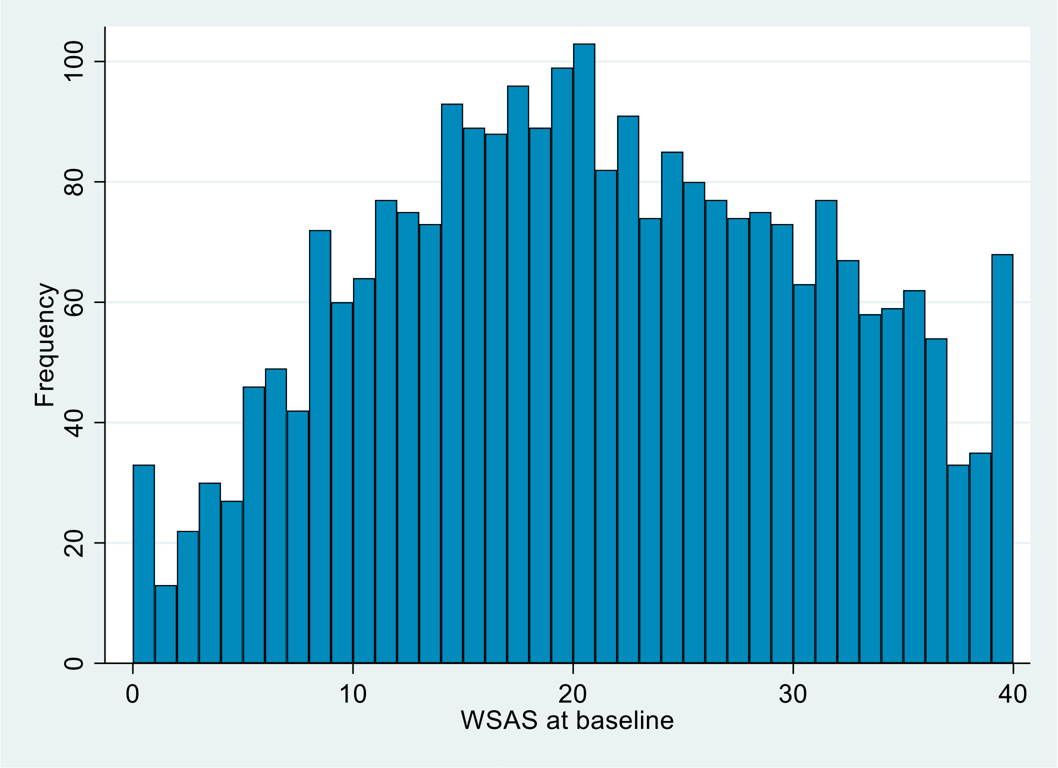

Appendix Figure 2: Frequency distribution of the first reported (baseline) EQ-5D Index Score

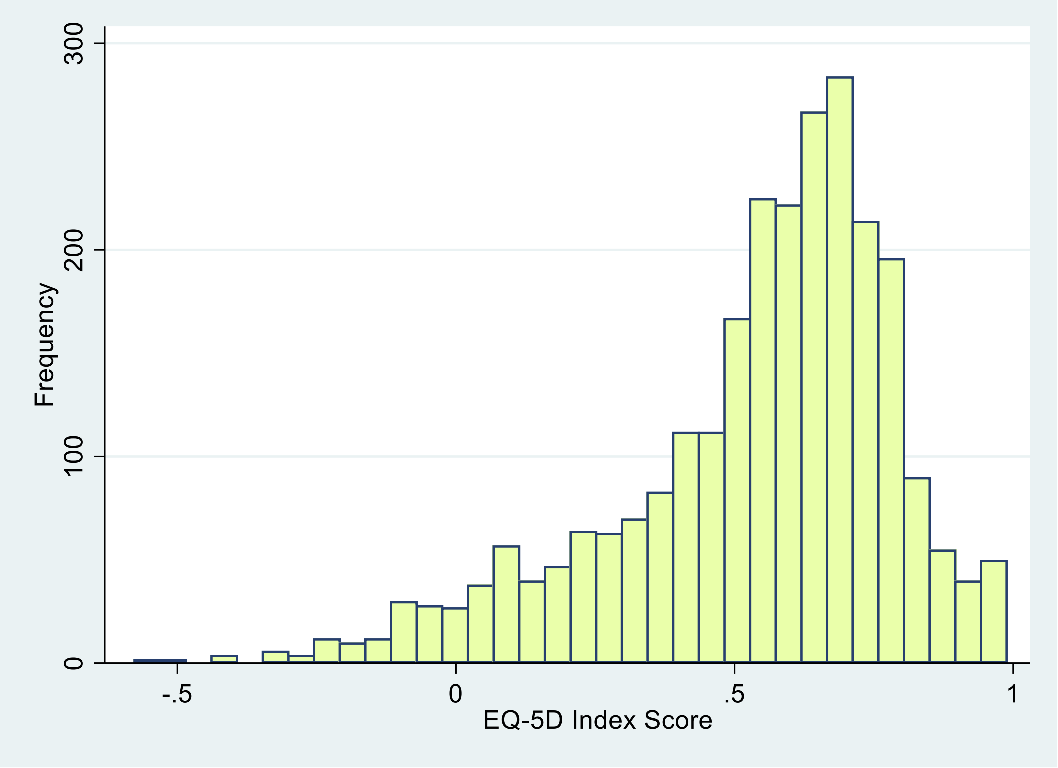

STROBE Statement—Checklist of items that should be included in reports of ***cross-sectional studies***

	Item No	Recommendation	Page No
**Title and abstract**	1	(*a*) Indicate the study's design with a commonly used term in the title or the abstract	1
		(*b*) Provide in the abstract an informative and balanced summary of what was done and what was found	3
**Introduction**			
Background/rationale	2	Explain the scientific background and rationale for the investigation being reported	5
Objectives	3	State specific objectives, including any prespecified hypotheses	6
**Methods**			
Study design	4	Present key elements of study design early in the paper	6
Setting	5	Describe the setting, locations, and relevant dates, including periods of recruitment, exposure, follow-up, and data collection	6-7
Participants	6	(*a*) Give the eligibility criteria, and the sources and methods of selection of participants	7
Variables	7	Clearly define all outcomes, exposures, predictors, potential confounders, and effect modifiers. Give diagnostic criteria, if applicable	7-8
Data sources/ measurement	8*	For each variable of interest, give sources of data and details of methods of assessment (measurement). Describe comparability of assessment methods if there is more than one group	8
Bias	9	Describe any efforts to address potential sources of bias	10
Study size	10	Explain how the study size was arrived at	7
Quantitative variables	11	Explain how quantitative variables were handled in the analyses. If applicable, describe which groupings were chosen and why	9-10
Statistical methods	12	(*a*) Describe all statistical methods, including those used to control for confounding	9-10
		(*b*) Describe any methods used to examine subgroups and interactions	N/A
		(*c*) Explain how missing data were addressed	10
		(*d*) If applicable, describe analytical methods taking account of sampling strategy	N/A
		(*e*) Describe any sensitivity analyses	N/A
**Results**			
Participants	13*	(a) Report numbers of individuals at each stage of study—eg numbers potentially eligible, examined for eligibility, confirmed eligible, included in the study, completing follow-up, and analysed	11
		(b) Give reasons for non-participation at each stage	N/A
		(c) Consider use of a flow diagram	N/A
Descriptive data	14*	(a) Give characteristics of study participants (eg demographic, clinical, social) and information on exposures and potential confounders	11-17
		(b) Indicate number of participants with missing data for each variable of interest	15-16
Outcome data	15*	Report numbers of outcome events or summary measures	15-16

For peer review only

For peer review only - http://bmjopen.bmj.com/site/about/guidelines.xhtml

Main results	16	(*a*) Give unadjusted estimates and, if applicable, confounder-adjusted estimates and their precision (eg, 95% confidence interval). Make clear which confounders were adjusted for and why they were included	19-20
		(*b*) Report category boundaries when continuous variables were categorized	15-16
		(*c*) If relevant, consider translating estimates of relative risk into absolute risk for a meaningful time period	19-20
Other analyses	17	Report other analyses done—eg analyses of subgroups and interactions, and sensitivity analyses	N/A
**Discussion**			
Key results	18	Summarise key results with reference to study objectives	23
Limitations	19	Discuss limitations of the study, taking into account sources of potential bias or imprecision. Discuss both direction and magnitude of any potential bias	24
Interpretation	20	Give a cautious overall interpretation of results considering objectives, limitations, multiplicity of analyses, results from similar studies, and other relevant evidence	24-25
Generalisability	21	Discuss the generalisability (external validity) of the study results	25
**Other information**			
Funding	22	Give the source of funding and the role of the funders for the present study and, if applicable, for the original study on which the present article is based	25

*Give information separately for exposed and unexposed groups.

Note: An Explanation and Elaboration article discusses each checklist item and gives methodological background and published examples of transparent reporting. The STROBE checklist is best used in conjunction with this article (freely available on the Web sites of PLoS Medicine at http://www.plosmedicine.org/, Annals of Internal Medicine at http://www.annals.org/, and Epidemiology at http://www.epidem.com/). Information on the STROBE Initiative is available at www.strobe-statement.org.

For peer review only - http://bmjopen.bmj.com/site/about/guidelines.xhtml

BMJ Open

The impact of fatigue as the primary determinant of functional limitations amongst patients with Post-COVID syndrome: a cross-sectional observational study

Journal:	*BMJ Open*
Manuscript ID	bmjopen-2022-069217.R1
Article Type:	Original research
Date Submitted by the Author:	28-Feb-2023

Complete List of Authors:	Walker, Sarah; University of Exeter, The Department of Health and Community Sciences (Medical School)<br>Goodfellow, Henry; University College London, Department of Primary Care and Population Health<br>Pookarnjanamorakot, Patra; Whittington Health NHS Trust, General Medicine<br>Murray, Elizabeth; University College London, Primary Care and Population Health<br>Bindman, Julia; University College London, Research Department of Primary Care and Population Health<br>Blandford, Ann; University College London, UCLIC, Dept of Computer Science<br>Bradbury, Katherine; University of Southampton, Psychology<br>Cooper, Belinda; University College London, Research Department of Primary Care and Population Health<br>Hamilton, Fiona; University College London, Research Department of Primary Care and Population Health<br>Hurst, John; University College London, UCL Respiratory<br>Hylton, Hannah; Barts Health NHS Trust, Department of Respiratory Medicine<br>Linke, Stuart; University College London, Research Department of Primary Care and Population Health<br>Pfeffer, Paul; Barts Health NHS Trust, Department of Respiratory Medicine<br>Ricketts, William; Barts Health NHS Trust, Respiratory Medicine<br>Robson, Chris; Living With Ltd, 10 Queen Street Place, London, EC4R 1AG<br>Stevenson, Fiona; University College London, Research Department of Primary Care and Population Health<br>Sunkersing, David; University College London, Research Department of Primary Care and Population Health<br>Wang, Jiunn; University College London, Department of Applied Health Research<br>Gomes, Manuel; University College London, Department for Applied Health Research<br>Henley, William; University of Exeter, The Department of Health and Community Sciences (Medical School)<br>Collaboration, LivingWith Covid Recovery ; University College London

**Primary Subject Heading**:	Public health
Secondary Subject Heading:	General practice / Family practice, Global health, Health policy, Health services research, Infectious diseases
Keywords:	COVID-19, Public health < INFECTIOUS DISEASES, MENTAL HEALTH, PRIMARY CARE, Anxiety disorders < PSYCHIATRY, Depression & mood disorders < PSYCHIATRY

SCHOLARONE™
Manuscripts

I, the Submitting Author has the right to grant and does grant on behalf of all authors of the Work (as defined in the below author licence), an exclusive licence and/or a non-exclusive licence for contributions from authors who are: i) UK Crown employees; ii) where BMJ has agreed a CC-BY licence shall apply, and/or iii) in accordance with the terms applicable for US Federal Government officers or employees acting as part of their official duties; on a worldwide, perpetual, irrevocable, royalty-free basis to BMJ Publishing Group Ltd ("BMJ") its licensees and where the relevant Journal is co-owned by BMJ to the co-owners of the Journal, to publish the Work in this journal and any other BMJ products and to exploit all rights, as set out in our licence.

The Submitting Author accepts and understands that any supply made under these terms is made by BMJ to the Submitting Author unless you are acting as an employee on behalf of your employer or a postgraduate student of an affiliated institution which is paying any applicable article publishing charge ("APC") for Open Access articles. Where the Submitting Author wishes to make the Work available on an Open Access basis (and intends to pay the relevant APC), the terms of reuse of such Open Access shall be governed by a Creative Commons licence – details of these licences and which Creative Commons licence will apply to this Work are set out in our licence referred to above.

Other than as permitted in any relevant BMJ Author's Self Archiving Policies, I confirm this Work has not been accepted for publication elsewhere, is not being considered for publication elsewhere and does not duplicate material already published. I confirm all authors consent to publication of this Work and authorise the granting of this licence.

For peer review only

\Title The impact of fatigue as the primary determinant of functional limitations amongst patients with Post-COVID syndrome: a cross-sectional observational study

Corresponding Author:

Professor William Henley, The Department of Health and Community Sciences (Medical School), University of Exeter, St Luke's Campus, Exeter, England, EX1 2LU

Email: W.E.Henley@exeter.ac.uk

Authors

Dr Sarah Walker, The Department of Health and Community Sciences (Medical School), University of Exeter, St Luke's Campus, Exeter, UK, EX1 2LU.

Henry Goodfellow, Research Department of Primary Care and Population Health, University College London, London, UK

Patra Pookarnjanamorakot, General Medicine, Whittington Health NHS Trust, London, UK

Elizabeth Murray, Research Department of Primary Care and Population Health, University College London, London, UK

Julia Bindman, Research Department of Primary Care and Population Health, University College London, London, UK

Ann Blandford, UCLIC, Dept of Computer Science, UCL, London WC1E 6BT, UK.

Katherine Bradbury, Psychology, University of Southampton, Southampton, UK

Belinda Cooper, Research Department of Primary Care and Population Health, University College London, London, UK

Fiona L Hamilton, Research Department of Primary Care and Population Health, University College London, London, UK

John R Hurst, UCL Respiratory, University College London, London, UK

Hannah Hylton, Department of Respiratory Medicine, Barts Health NHS Trust, London, UK

Stuart Linke, Camden and Islington Mental Health Trust and Research Department of Primary Care and Population Health, University College London, London, UK

Paul E Pfeffer, Department of Respiratory Medicine, Barts Health NHS Trust, London, UK and Barts and The London School of Medicine and Dentistry, Queen Mary University of London, London, UK.

William Ricketts, Department of Respiratory Medicine, Barts Health NHS Trust, London, UK

Chris Robson, Living With Ltd, London, UK

Fiona A Stevenson, Research Department of Primary Care and Population Health, University College London, London, UK

For peer review only

David Sunkersing, Research Department of Primary Care and Population Health, University College London, London, UK

Jiunn Wang, Department of Applied Health Research, University College London, London, UK

Manuel Gomes, Department for Applied Health Research, University College London, London, UK

William Henley, The Department of Health and Community Sciences (Medical School), University of Exeter, St Luke's Campus, Exeter, UK, EX1 2LU.

On behalf of the Living With Covid Recovery Collaboration

Word count 4770

Keywords

Fatigue, functional limitation, Long-COVID, Post-COVID, COVID-19, WSAS, SARS-CoV-2, EQ-5D, HRQoL

For peer review only

ABSTRACT

Objectives

To describe self-reported characteristics and symptoms of treatment-seeking Post-COVID Syndrome (PCS) patients. To assess the impact of symptoms on health-related quality of life and patients' ability to work and undertake activities of daily living.

Design

Cross-sectional single-arm service evaluation of real-time user data.

Setting

31 Post-COVID clinics in the UK.

Participants

3,754 adults diagnosed with PCS in primary or secondary care, deemed suitable for rehabilitation.

Intervention

Patients using the Living With Covid Recovery (LWCR) Digital Health Intervention (DHI) registered between 30/11/20 and 23/03/22.

Primary and secondary outcome measures

The primary outcome was the baseline Work and Social Adjustment Scale (WSAS). WSAS measures the functional limitations of the patient; scores ≥20 indicate moderately severe limitations. Other symptoms explored included fatigue (FACIT-F), depression (PHQ-8), anxiety (GAD-7), breathlessness (MRC Dyspnoea Scale and Dyspnoea-12), cognitive impairment (PDQ-5) and health-related quality of life (EQ-5D). Symptoms and demographic characteristics associated with more severe functional limitations were identified using logistic regression analysis.

Results

3541 (94%) patients were of working age (18-65); mean age (SD) 48 (12) years; 1282 (71%) were female and 89% were White. 51% reported losing ≥1 days from work in the previous 4 weeks; 20% reported being unable to work at all. Mean WSAS score at baseline was 21 (SD 10) with 53% scoring ≥20. Factors associated with WSAS scores ≥20 were high levels of fatigue, depression and cognitive impairment. Fatigue was found to be the main symptom contributing to a high WSAS score.

Conclusions

A high proportion of this PCS treatment-seeking population was of working age with over half reporting moderately severe or worse functional limitation. There were substantial impacts on ability to work and activities of daily living in people with PCS. Clinical care and rehabilitation should address the management of fatigue as the dominant symptom explaining variation in functionality.

(299 words)

For peer review only - http://bmjopen.bmj.com/site/about/guidelines.xhtml

Stengths and Limitations of this study

- Large cohort of patients (n=3754) with novel disease from 31 specialised Post-COVID clinics in England and Wales.
- Patient Reported Outcome Measures (PROMs) contain 8 validated questionnaires including common Post-COVID Syndrome (PCS) symptoms, quality of life (EQ-5D) and functional status (WSAS), allowing comparison with other health conditions.
- High completion rate of PROMs at baseline (registration) ensures reported data is representative of LWCR DHI users
- As data was collected through a Digital Health Intervention (DHI), some clinical data on PCS patients was not available, such as date of acute COVID infection(s) and vaccination status.
- Regression analysis was used on available data; we acknowledge that missing data may have introduced bias.

For peer review only

For peer review only - http://bmjopen.bmj.com/site/about/guidelines.xhtml

INTRODUCTION

Post-COVID Syndrome (PCS), or “Long-COVID”, is defined by National Institute for Health & Care Research (NIHR) and the World Health Organization (WHO) as the signs and symptoms of the disease that continue for more than 12 weeks after the initial acute covid infection. [1]It is causing increasing concern due to the potential number of patients infected and the associated morbidity caused by the symptoms.

As of the 2nd August 2022, there have been over 577 million cases of COVID-19 worldwide. [2] There have been various estimates on the number of patients with acute COVID-19 that go on to develop PCS, ranging from 3.0% to 14.1% [1, 3-6] with over 1.4 million people in the UK reporting PCS symptoms as of July 2022. [6] The symptoms of PCS include fatigue, breathlessness, brain fog, anosmia, and mental health problems. These symptoms can cause debilitating functional and psychological limitations [3, 7]and have been shown to persist for up to two years. [1, 3, 6, 8-10] This has led to many people with PCS being unable to work or care for others for a prolonged period. [7] The potential impact of PCS on national health services, economies and population health is attracting international attention as the associated morbidity and economic effects become clearer. [5, 11-17]

The UK National Health Service (NHS) has set up Post-COVID Assessment Clinics to provide care for the large number of patients with PCS . [6, 18] In the absence of pharmacotherapies shown to be effective for this condition, management of people with PCS has to date focused on self-management education and rehabilitation programmes. These clinics provide specialist rehabilitation from a range of health care professionals including respiratory specialist doctors, GPs, Physiotherapists, Occupational Therapists and Psychologists. Over 30 of these clinics were augmented with a bespoke Digital Health Intervention (DHI), called Living With Covid Recovery, to enable remote rehabilitation for PCS patients during the COVID-19 pandemic. Internationally, despite the growing number of PCS patients, the strategies to combat PCS are at their early stages with no standard rehabilitation pathway. [11-14] As the pandemic continues, PCS will continue to add significant workload for health services beyond acute COVID-19 care. [19]

This study is the first to present the baseline symptoms and functional impairment from a treatment-seeking PCS population across multiple centres and to estimate the contribution of different patient-reported symptoms to impairment. These data will help clinicians and policy makers plan appropriate services.

METHODS

Design and setting

Cross-sectional observational study of patients using the Living With Covid Recovery Digital Health Intervention as part of their assessment and treatment in 31 self-selecting specialised Post-COVID clinics in England and Wales.

Intervention

Living With Covid Recovery (LWCR) is a bespoke Digital Health Intervention (DHI), designed to be part of Post-COVID Clinics. The LWCR DHI was designed by a multi-disciplinary team of

For peer review only - http://bmjopen.bmj.com/site/about/guidelines.xhtml

clinicians, Patient and Public Involvement (PPI), academics and industry partners. [20] The product was first launched in a clinical setting in August 2020 and since then has been updated 8 times. The DHI contains 12 (8 validated) patient-reported outcome measures (PROMs) in the form of validated questionnaires completed by patients as part of their clinical care. In this study, we use 10 of these (8 validated). Six are related to symptoms and one related to each of patient demographics (unvalidated), functional ability, quality of life and health service use (unvalidated). More details are provided in the 'Patient Reported Outcome Measures (PROMs)' section below and in the study protocol. The Work and Social Adjustment Scale (WSAS) questionnaire was introduced in February 2021 and the Demographic questionnaire in April 2021. Development followed the principles of human computer interaction agile development, with updates to the DHI based on feedback from healthcare practitioners and our PPI group. All data collected in the LWCR product were pseudo-anonymised, using a unique patient ID number and stored in Metabase (www.metabase.com).

Population

Patients included in this study were those who had registered to use the LWCR DHI as part of the clinical care provided in a Post-COVID Syndrome NHS community clinic in England and Wales. Patients are referred to these clinics from Primary or Secondary Care after having experienced Post-COVID symptoms for 12 weeks or more.

Eligible patients were identified as being suitable for remote rehabilitation service by the clinic if they were aged 18 or over, had access to a smart phone device, were considered likely to benefit from the intervention, fit for rehabilitation and were able to read English. Patients registered on the LWCR DHI between 30/11/20 and 23/03/22.

Outcomes

Primary Outcome

The Work and Social Adjustment Scale was the primary outcome measure for this study. WSAS is a validated questionnaire for functional impairment. [21] Scores range between 0 and 40, with scores of 20 or more indicating moderately severe or worse impairment on daily functioning. [21] The WSAS contains 5 equally weighted component scores (range 0 to 8), relating to impairments across the following domains:

1) Ability to work
2) Home management
3) Social leisure activities
4) Private leisure activities
5) Close relationships

Additionally, there is a further question to identify those individuals who are either retired or have chosen not to work. There is no defined recall period for the WSAS, therefore the questionnaire reflects the currrent situation.

Secondary Outcome

For peer review only - http://bmjopen.bmj.com/site/about/guidelines.xhtml

The secondary outcome was the EQ-5D, a standardised measure of health-related quality of life. [22] The EQ-5D-5L descriptive system comprises five dimensions (mobility, self-care, usual activities, pain / discomfort, and anxiety / depression). For each dimension, there are 5 possible responses (level 1: no problems, level 2: slight problems, level 3: moderate problems, level 4: severe problems, level 5: unable to/extreme problems). The responses are coded to give a 5-digit code to describe the respondent's health state (such as 13254). Reference weights from the UK general population are applied to the resulting health states to produce a single summary index score for health status, the EQ-5D-5L index score. This is a measure anchored at 0 (representing 'death') and 1 ('full health'), but it can include negative values to reflect health states judged worse than death. Similar to the WSAS, there is no recall period defined for the EQ-5D, therefore the PROM would reflect the health status on the day of questionnaire completion.

Explanatory Variables

Patient Demographics

The data collected in the Patient Demographic Questionnaire included patient reported age, gender, ethnicity, highest level of education and postcode. Patient age and gender were also reported by the clinic when registering the patient to use the DHI. Early versions of the DHI did not include the demographic questionnaire, which became available to all patients in April 2021. Where both clinic and patient-reported data were available, patient-reported age, gender and ethnicity were used, with clinic-reported data used as back-up.

To keep the data pseudo-anonymised, the Index of Multiple Deprivation (IMD) was provided to the study statistician, rather than the patient postcode. The English Indices of Deprivation (2019) was used to provide the IMD from the patient's postcode. [23] The IMD decile was not provided for 35 patients who had completed the demographic questionnaire. These were either entered incorrectly or were new, so not in the latest update of the IMD registry. Additionally, patient date of birth (as supplied by the clinic) was replaced with year of birth, from which an approximate age could be calculated.

Patient Reported Outcome Measures (PROMs)

In this study, six validated questionnaires were used to capture the severity of five of the core symptoms of PCS through patient-reported outcome measures (PROMs). The PROMs were completed by patients based on their clinical need, as determined by the patient themselves or with their health care professional. The first PROM completed by the patient was taken as their baseline measurement. The date and time of completion in relation to when the patient first registered to use the DHI was recorded, along with the outcome scores. PROMs were analysed as continuous variables, unless stated otherwise. Where threshold values for caseness are available, we present the number of patients within each of these categories to enable comparison between this study and other research.

1. Breathlessness

a) Dyspnoea-12 gives an overall score of breathlessness impact, with higher scores corresponding to greater severity. [24-26] [Recall period not defined, reflects current moment]

For peer review only - http://bmjopen.bmj.com/site/about/guidelines.xhtml

b) MRC Dyspnoea Scale measures the degree of breathlessness related to activity, with higher scores corresponding to greater severity. [27-28] The scale takes the values 1 to 5, using the following classifications: MRC 1 (Mild); MRC 2 to 3 (Moderate) and MRC 4 to 5 (Severe). [29] We analysed this variable as a categorical score. [Recall period not defined, reflects current moment]

2. Fatigue
   Functional Assessment of Chronic Illness Therapy – Fatigue (FACIT-F) measures self-reported fatigue and its impact on daily activities and function with lower scores corresponding to greater fatigue. A threshold value of 30 was chosen in line with fatigue reported in a cancer population. [26] Population mean value for FACIT-F in the general population has been reported as 43. [25, 26, 30] [Recall period: 7 days]

3. Anxiety
   The Generalized Anxiety Disorder scale (GAD-7) is used as a screening tool and severity measure for anxiety. [31]A cut off value of 10 or more identifies anxiety. Additionally, threshold values are also considered: No anxiety (0-4); Mild anxiety (5 to 9); Moderate anxiety (10 to 14) and Severe anxiety (15 to 21). [Recall period: 2 weeks]

4. Cognition (brain fog)
   The Perceived Deficits Questionnaire, 5 item version (PDQ-5) measures the degree to which individuals perceive themselves as experiencing cognitive difficulties. [32-33] Higher scores indicate more perceived deficits. The following threshold values suggested by Lam [34] are used: Minimal 0-8; Moderate 9-14; Severe 15-20. [Recall period: 4 weeks]

5. Depression
   The Patient Health Questionnaire eight item depression scale (PHQ-8) was chosen over the 9-item (PHQ-9) PROM for this study as it was not always certain that adequate intervention would be available if the question on suicidal thoughts or self-harm was endorsed; therefore, this question was omitted. [35] The same scoring thresholds are used as for PHQ-9, with a score of 10 or more used as a cut off for a diagnosis of depression. [36] [Recall period: 2 weeks]

Statistical Analysis

Primary Outcome

Logistic regression was used to identify the PROMs associated with a high WSAS score (≥20) after accounting for the effects of demographic variables. First, we built a model for the demographic factors associated with high WSAS score. Age and gender were included as covariates in all models. Other demographics, including highest level of education, ethnicity (as white or non-white) and IMD quintile, were added using a stepwise approach based on the Likelihood Ratio (LR) Test. Any demographic variables with a p-value below 0.2 were retained for inclusion in subsequent models. At each stage, the McKelvey and Zavoina's R-

For peer review only - http://bmjopen.bmj.com/site/about/guidelines.xhtml

squared value of the model including the additional term was calculated as a measure of the proportion of variation in the binary WSAS outcome attributable to the selected factors. [37]

The FACIT-F score was reversed (calculated as 52 minus reported score), to align the direction of the score with other variables in the analysis. Higher values of the score now represent greater fatigue. We refer to this as FACIT-F (reversed scale).

Next, we added each of the PROMs (Dyspnoea-12, MRC-Dyspnoea, FACIT-F (reversed scale), GAD-7, PDQ-5, and PHQ-8) in a univariable fashion to the logistic regression model for the demographic factors. Any PROMs with a p-value below 0.2 were retained for potential inclusion in subsequent models. A multivariable model including both demographics and PROMs was developed by sequentially adding or removing PROMs according to the LR test using a p-value threshold of 0.05. The McKelvey and Zavoina's R-squared value was calculated at each stage as a measure of model fit. For the final model, we calculated the reduction in R-squared from removing each PROM from the model as a measure of the contribution of that variable to explaining variance in the WSAS outcome. Standardised effect estimates were produced to facilitate comparisons between the effect sizes of the PROMs, as they were each measured on different scales.

The analysis was conducted using a complete cases approach, assuming data were missing at random (MAR) conditional on the variables included in the regression models. Comparisons were made between the demographic characteristics of the full sample of treatment-seeking patients and those providing a baseline WSAS measure to assess the potential for selection bias due to the exclusion of patients with missing WSAS scores.

Secondary Outcomes

WSAS Domain score analysis

Secondary analysis was conducted to assess the extent to which the PROMs identified in the main analysis were associated with the individual domain scores of each of the 5 WSAS domains. The PROMs used in the multivariable logistic model were tested as explanatory variables in linear regression models for each of the 5 domains of ability to work, home management, social leisure activities, private leisure activities and close relationships. Models were adjusted for age and gender as in the primary analysis. Standardised estimates of effect size and change in adjusted R-squared values were calculated for each PROM in the multivariable model.

EQ-5D-5L analysis

Frequencies and proportions of patients reporting each dimension and level of EQ-5D-5L were calculated. Linear regression analysis of the EQ-5D index score was carried out to quantify the effect of patient demographics and PROMs on health-related quality of life (HRQoL). Multivariable linear regression models for the EQ-5D-5L analysis were developed adopting the same model selection strategy used in the primary analysis.

Working days lost due to Post-COVID syndrome

For peer review only - http://bmjopen.bmj.com/site/about/guidelines.xhtml

Additionally, LWCR users were asked to complete a study-specific questionnaire to capture data on the number of working days lost in the 28 days prior to questionnaire completion. Users were asked "In the last 4 weeks how many days off work (sick leave) have you taken due to Covid-19 and/or rehabilitation." The correlation between the number of working days lost and the WSAS 'work' domain was estimated.

All analyses were carried out in Stata version 17.0.

Patient and Public Involvement

This study had substantial PPI involvement with co-investigator (JB), steering group (JB, KB), individual work package management groups and an overall PPI Advisory Group. The feedback from PPI at an early stage was essential in determining the PROMs chosen in the study and the primary outcome measure of the WSAS. [20]

RESULTS

Patient Demographics

The study included 3754 treatment-seeking PCS patients with a mean age of 47.7 (SD 12.3) years, and 3541 (94.4%) being of working age (18–65) from across 31 clinics in the UK. The population were 71% (n=2675) female and 87% (n =2414) of White ethnicity (Table 1) and skewed toward affluence, with 11% (n =289) from the most deprived quintile and 24% (n=642) from the least deprived. Just over a half (n=1466, 53%) were educated to degree level or higher. Similar patient characteristics were seen in those who completed the WSAS and EQ-5D PROMs compared to the overall sample of patients using the app (Table 1).

For peer review only

For peer review only - http://bmjopen.bmj.com/site/about/guidelines.xhtml

Table 1: Sociodemographic characteristics of the patients in the study

Patient characteristic<br>n (%) unless stated otherwise	Study population<br>n (%)<br>(N=3754)	WSAS completed<br>n (%)<br>(n=2627)	EQ-5D-5L completed<br>n (%)<br>(n=2643)
Age (years), mean (SD)	47.7 (12.3)<br>(n=3753)	47.2 (11.9)	47.2 (11.9)
Age category (years)			
18 – 29	349 (9.3)	236 (9.0)	237 (9.0)
30 – 39	615 (16.4)	439 (16.7)	440 (16.6)
40 – 49	1084 (28.9)	771 (29.3)	773 (29.2)
50 – 59	1127 (30.0)	815 (31.0)	820 (31.0)
60 – 69	469 (12.5)	310 (11.8)	317 (12.0)
70 and over	109 (2.9)	56 (2.1)	56 (2.1)
Missing*	1	0	0
Gender			
Female	2675 (71.3)	1898 (72.3)	1909 (72.3)
Male	1060 (28.2)	719 (27.4)	724 (27.4)
Non-binary	10 (0.3)	9 (0.3)	9 (0.3)
Missing*	9	1	1
Highest Educational Level			
No education	113 (4.1)	106 (4.1)	102 (4.0)
School leaver (NVQ 1-2)	611 (22.1)	574 (22.5)	574 (22.6)
A-Level (NVQ-3)	574 (20.8)	532 (20.8)	533 (21.0)
Degree (NVQ-4)	581 (21.0)	527 (20.6)	526 (20.7)
Postgraduate Degree (NVQ-5)	885 (32.0)	817 (32.0)	808 (31.8)
Missing*	990	71	100
Ethnicity			

For peer review only - http://bmjopen.bmj.com/site/about/guidelines.xhtml

White	2414 (87.3)	2242 (87.7)	2234 (87.8)
Asian or Asian British	177 (6.4)	159 (6.2)	155 (6.1)
Black African Caribbean or Black British	55 (2.0)	48 (1.9)	47 (1.8)
Mixed or Multiple Ethnicity	67 (2.4)	61 (2.4)	62 (2.4)
Other ethnic group	32 (1.2)	27 (1.1)	26 (1.0)
Prefer not to say	19 (0.7)	19 (0.7)	19 (.7)
Missing*	990	71	100
IMD Quintile			
1 to 2 (20 % most deprived)	289 (10.6)	274 (10.9)	272 (10.8)
3 to 4	537 (19.7)	500 (19.8)	491 (19.6)
5 to 6	657 (24.1)	610 (24.2)	606 (24.1)
7 to 8	604 (22.1)	555 (22.0)	556 (22.1)
9 to 10 (20% least deprived)	642 (23.5)	585 (23.2)	586 (23.3)
Missing*	1025	103	132

* Data on patient-reported characteristics is missing for 990 who did not complete the Patient Demographics questionnaire. In addition, a further 35 are missing IMD as their IMD decile was not available. Percentages do not include those with missing values in the denominator

For peer review only

The functional impairment and quality of life of the treatment seeking PCS population

Functional impairment

Characteristics of patients who completed the WSAS PROM were similar to those of all users of the LWCR DHI (Table 1). The population reported a very high degree of functional impairment (mean WSAS score of 20.6, n=2627), with over half the patients (53%) scoring above 20 in the moderately severe category (Appendix 1, Appendix Figure 1). Functional impairment was seen across all five of the WSAS domains; with the highest rates of functional impairment seen in the Social Leisure Activities and Ability to Work categories; mean scores 4.7 and 4.6, respectively. The least affected domain in PCS patients was close relationships with a mean score of 3.0 (Appendix 1). Ethnicity was not a contributing factor to the WSAS score; ethnicity was not significant in the univariable analysis and was therefore dropped from subsequent models. In increasing order, the mean WSAS score across the ethnic groups was: Mixed or multiple ethnic groups: 9.7; White: 9.8; Asian or Asian British: 10.4; Other ethnic group: 10.4; Black, Black British, Caribbean or African 10.7 and 12.8 in those who preferred not to provide their ethnicity.

Health related quality of life

EQ-5D data was completed by 2643 LWCR DHI users. Patients reported a large impact on health-related quality of life, with an average (median) EQ-5D index score of 0.60 (IQR 0.41 to 0.71) (Appendix Figure 2).

Appendix 2 shows the number of respondents reporting a problem in each domain. The two domains of the EQ-5D most affected by PCS were pain/discomfort reported by 2542 (96.2%) and anxiety/depression reported by 2509 (95%). The least affected EQ-5D domain was usual activities, with 36% reporting no problems.

Working days lost due to Post-COVID syndrome

Half (n=1321/2600, 50.8%) of patients who completed the study-specific questionnaire reported losing one or more days from work in the previous month, with a fifth (20.3%) reporting between 20 and 28 working days lost. (Appendix 3) Correlation between the baseline WSAS work domain (score 0 to 8) and number of working days lost was 0.52, showing moderate correlation.

Severity of patient reported symptoms

The LWCR DHI users were extremely fatigued, reporting a mean FACIT-F score of 19.6, well below the threshold value of 30 used in this study. (FACIT-F reversed scale mean 32.4; threshold value of 22). Mental health was affected, with a mean GAD-7 score of 9 (corresponding to mild anxiety) and a mean PHQ-8 of 11.8, meeting the clinical threshold for depression. Additionally, breathlessness was evident, with a mean Dyspnoea-12 score of 12 and median (IQR) MRC Dyspnoea Scale score of 2 (2,3). The PCS population also reported moderate cognitive difficulties (brain fog) with a mean PDQ-5 score of 12. (Table 2).

For peer review only - http://bmjopen.bmj.com/site/about/guidelines.xhtml

For peer review only

For peer review only - http://bmjopen.bmj.com/site/about/guidelines.xhtml

Table 2: Summary of Patient Reported Outcome Measures (PROMs) and scores for users of the Living With Covid Recovery DHI. Overall mean (SD) and number (%) within each threshold category are reported.

PROM	Measures	Number completed	Mean (SD)	Threshold values [Number in each threshold category (%)]
Work and Social Adjustment Score (WSAS) ***Primary Outcome***	Functional limitations of the patient. Higher scores indicate greater functional impairment. Range:0-40	2627	20.6 (9.9)	<10: subclinical [394 (15.0)]<br>10 – 19: significant [843 (32.1)]<br>>20: Moderately severe [1390 (52.9)]
Ability to work*	Functional limitations within domains. Subscale range: 0-8 0: not at all affected to 8: very severely affected.	2621	4.6 (2.4)	
Home management		2627	4.2 (2.2)	
Social leisure activities		2627	4.0 (2.2)	
Private leisure activities		2627	4.7 (2.3)	
Close relationships		2627	3.0 (2.4)	
EQ-5D (EQ-5D-5L) ***Secondary Outcome***	A standardised measure of health status. Index scores range from 0 (equivalent to dead) to 1 (full health); negative values are possible.	2633	0.54 (0.27) Median: 0.60 (IQR 0.41 to 0.71)	
**Explanatory variables**				

For peer review only - http://bmjopen.bmj.com/site/about/guidelines.xhtml

Functional Assessment of Chronic Illness Therapy – Fatigue (FACIT-F)	Self-reported fatigue and its impact upon daily activities and function. Higher scores indicate less fatigue. Range: 0-52.	2890	19.6 (10.1)	<30: Impairment [2418 (83.7)]<br>≥30: No impairment [472 (16.3)]
FACIT-F (reversed scale)<br>Scale reversed in results to aid interpretation	Higher scores indicate greater fatigue. Range: 0-52.	2890	32.4 (10.1)	≤22: No impairment [472 (16.3)]<br>>22: Impairment [2418 (83.7)]
Generalized Anxiety Disorder scale (GAD-7)	Screening tool and severity measure for anxiety. Range: 0-21.	2774	9.0 (5.9)	<4: No anxiety [715 (25.8)]<br>5-9: Mild anxiety [870 (31.4)]<br>10-14: Moderate anxiety [591 (21.3)]<br>≥15: Severe anxiety [598 (21.6)]
Patient Health Questionnaire depression scale (PHQ-8)	A valid diagnostic and severity measure for current depressive disorders. Range: 0-24.	2661	11.8 (6.0)	<10: No depression [1034 (38.9)]<br>≥10: Clinical depression [1627 (61.1)]
Dyspnoea-12	Overall score of breathlessness impact, with higher scores corresponding to greater severity. Range: 0 to 36.	2656	12.0 (9.3)	No threshold values
MRC Dyspnoea Scale (Median (IQR))	Degree of breathlessness related to activity. Range: 1 to 5.	2607	2 (2,3)	1: Mild [262 (10.1)]<br>2-3: Moderate [1800 (69.0)]<br>4-5: Severe [545 (20.9)]
Perceived Deficits Questionnaire, 5 item version (PDQ-5)	Degree to which individuals perceive themselves as experiencing cognitive difficulties. Range: 0-20.	2783	12.3 (4.3)	≤8: Minimal [519 (18.7)]<br>9-14: Moderate [1346 (48.4)]<br>≥15: Severe [918 (33.0)]

* Reduced number of completed answers as patients who had retired or chose not to work did not need to answer this question.

For peer review only - http://bmjopen.bmj.com/site/about/guidelines.xhtml

Contribution of Fatigue to functional impairment and health related quality of life

Functional impairment

Fatigue, depression, and cognitive impairment were significant predictors of a high WSAS (functional impairment) score. Fatigue was the strongest predictor of high WSAS, with a one-point increase in the reversed FACIT-F associated with an increase of 16% in the odds of a patient having a high WSAS score. When sequentially removing each PROM from the final multivariable model, the greatest contribution to reduction in R-squared (measure of goodness of fit of the statistical model) was attained by the removal of FACIT-F (33.8%), compared to a 1.7% reduction in R-squared for both PHQ-8 and PDQ-5) (Table 3).

Figure 1a shows the heat map distribution of WSAS scores with almost all the high scores (denoted by pink squares) above the FACIT-F threshold for impairment. In contrast, the high WSAS scores are spread more evenly across both sides of the cognition and depression threshold of 10 for PDQ-5 and PHQ-8 respectively (Figures 1a and 1b). FACIT-F also contributed strongly to the scores for each of the five WSAS domains, with PHQ-8 only making a substantive contribution, outperforming that of FACIT-F, in the 'close relationships' domain. The contribution of PDQ-5 was small compared to FACIT-F, with ability to work most associated with cognition. (Figure 2).

There was no significant difference in the functional impairment between genders, but a higher rate of functional impairment was seen in the younger age groups. The highest rate was seen in the 30-39 age group, compared to the reference age category of age 18 to 29 (OR 1.18, 95% CI 0.78 to 1.77; Table 3).

For peer review only - http://bmjopen.bmj.com/site/about/guidelines.xhtml

Table 3: WSAS multivariable model for different patient characteristics and PROM scores (N=2556)

Patient Characteristics		Odds Ratio (95% CI)	p-value	Reduction in R-squared [Full model R^2= 0.529]	Standardised effect size
**Age**	18 – 29	Reference			
	30 – 39	1.18 (0.78, 1.77)	0.441		
	40 – 49	0.90 (0.62, 1.32)	0.603		
	50 – 59	0.62 (0.42, 0.90)	0.011		
	60 – 69	0.55 (0.35, 0.85)	0.008		
	70 and over	0.26 (0.12, 0.59)	0.001		
**Gender**	Male	Reference			
	Female	0.83 (0.66,1.05)	0.115		
	Non-binary	0.25 (0.05, 1.17)	0.078		
**PROMs**	FACIT-F (reversed scale) High values indicate greater fatigue	1.16 (1.14, 1.18)	<0.001	0.179	4.47
	PHQ-8 High values indicate more severe depression	1.05 (1.03, 1.08)	<0.001	0.009	1.37
	PDQ-5	1.06 (1.03, 1.09)	<0.001	0.009	1.29

For peer review only - http://bmjopen.bmj.com/site/about/guidelines.xhtml

	High values indicate more perceived deficits				

For peer review only - http://bmjopen.bmj.com/site/about/guidelines.xhtml

Health related quality of life

Fatigue also contributed to the health-related quality of life of PCS patients with the FACIT-F (reversed scale) being a significant predictor of the EQ-5D index score. FACIT-F (reversed scale) made the largest contribution to explaining variation in quality of life (change in R-squared of 8.4% compared to 5.6% for MRC Dyspnoea Scale, 3.1% for GAD-7, 1.7% for PHQ-8 and 0.5% for Dyspnoea-12. (Appendix 1).

DISCUSSION

Principal findings

Treatment seeking Post-COVID patients consisting of mainly female, white, working age, and well-educated people are experiencing striking levels of functional impairment and low health-related quality of life. This impairment is mainly driven by their fatigue level, causing significant impact on their ability to work and care for others.

The patients report levels of functional impairment worse than in several other known clinical cohorts, such as patients referred to IAPT services in the South West of the UK (mean score 18.8 at referral). [38] Functional impairment was worse than in stroke patients (mean WSAS scores of 16) and comparable to patients with Parkinson's Disease (the mean WSAS scores ranged from 22.9 to 24.8), both debilitating neurological conditions. [39] Similarly, these patients report low Health-Related Quality of Life (HRQoL), with a mean EQ-5D score of 0.54 (SD 0.26), which compares poorly with patients with advanced/metastatic cancers. [40-41] For example, mean EQ-5D for stage IV lung cancer was between 0.66 and 0.84.[41] The results of the multivariable analysis show that fatigue is the strongest predictor of functional impairment (Table 3) and health-related quality of life (Appendix 4). Our population of patients reported worse fatigue (mean score of FACIT-F 19.6) than patients with stroke (mean score 38), inflammatory bowel disease (mean score 38.9), end stage renal disease (mean score 39) and even anaemic cancer patients (mean score 24) [30, 42-45] As well as patients reporting severe fatigue, they also report breathlessness, anxiety, depression and cognitive dysfunction.

This study is, to the best of our knowledge, the first reporting on functional limitations and health related quality of life in PCS from a national population of patients referred for specialist rehabilitation. As such, they differ from other cohort studies, which have followed up patients initially identified as hospitalised acute COVID patients (mean FACIT-F score 16.8) or through positive COVID testing in the general public. [46] One study has recently reported on a single centre Post-COVID assessment clinic showing similar levels of fatigue, but using a different measure (mean Fatigue Assessment Scale score 29) and inability of patients to work across 20hospitalised and non-hospitalised patients. [47] None of the other studies have reported on functional impairment using the WSAS which measures the impact PCS is having on patients' normal daily activities.

This study enforces the recommendation for the use of a consistent set of outcome measures in studies in COVID-19. One such list of recommended variables is the ICHOM Set of Patient-Centered Outcome Measures for COVID-19 which recommends that research assesses functional status, quality of life and social functioning in addition to the typically

For peer review only

reported measures of clinical outcomes, mental functioning, and symptom reporting. [48-49]Additionally, consideration should be given to the interpretation of fatigue in PCS patients, as advised by Sandler et al. [10] Patients may report fatigue when experiencing weakness, dyspnoea, cognitive dysfunction, somnolence or low mood.

Strengths and limitations of this study

All the data collected in this study were recorded in real time by patients and used by clinicians in their assessment and treatment. All PROMs used in the LWCR study were validated measures selected to provide the most reliable clinical information for patient benefit. Using these outcome measures allowed patient scores to be compared across disease types and with scores from other COVID studies. This necessity for clinically led data collection led to substantial missing data, partly due to the DHI evolving to include new features over the reported period; patients who used the DHI later in its development were able to complete more PROMs. The primary reason for App usage and associated data collection was not for research – as a result data on the severity of the initial disease or COVID-19 vaccination status were not collected within the app. Other studies have reported on the inconsistent relationship between severity of initial disease and severity of PCS, [46, 50] therefore we did not seek to capture further patient data from other sources.

Our chosen approach to the regression analysis was to use the observed data (a complete cases approach) but we acknowledge that exclusion of the missing data may have introduced bias. An alternative approach to analysing data that are missing at random would be to use multiple imputation but it has been recommended that complete cases analysis can be used as the primary analysis in situations where missing data is restricted to the dependent variable (we found very low levels of missing data in the explanatory variables when excluding patients with missing outcome data) and auxiliary variables have not been identified. [51]

Patients recruited to this study were sampled from the 31 specialist Post-COVID clinics that had chosen to use the LWCR DHI at the time of data extraction. Our sample is representative of the patients who are seen in PCS clinics nationally. The data may not be representative of all patients with Long COVID or PCS as many of these patients are not seen in a PCS clinic for a variety of reasons. This can be noted in the patient demographics which shows that the majority of our patients are white, affluent, and well-educated people. These patients are more likely to seek, and obtain, help than their counterparts.

This study has implications for the targeting of limited resources to effectively address functional limitations from PCS. Of particular concern is the large proportion of working age women in our study population, people who contribute substantially to the health care, social care and informal care sectors [52] at a time when these sectors are already under duress. [53] Post-COVID syndrome is clearly a multifactorial disease affecting physical and mental wellbeing but Post-COVID assessment services should consider focusing on assessing and treating fatigue to maximise the recovery and return to work in this large cohort of patients. Further work is needed to explore the recovery trajectories of this cohort over time and whether fatigue continues to predict functional impairment and low health-related quality of life over time.

For peer review only

CONCLUSION

In this first UK national study reporting clinical symptoms from patients referred for assessment and treatment of Post-COVID syndrome, we demonstrate high levels of functional impairment and low health-related quality of life. Fatigue appears to be the symptom most strongly associated with functional impairment. Currently, clinical services lack evidence-based approaches in treating patients experiencing fatigue related to PCS with no standard rehabilitation pathway. [11-14] This requires further targeted research. Our future work to explore the recovery trajectory of patients using the LWCR DHI may help to establish the extent to which WSAS, and other PROMs are sensitive to changes in the health of a patient with PCS. This work can contribute to the identification of PROMs best suited for use in assessing, managing, and treating patients with PCS, both digitally and in face-to-face appointments.

Contributorship statement

EM and HG were responsible for the concept of the Living With Covid Recovery study. HG is the guarantor. SW was the first author of the manuscript and revised it after review from the wider study team. SW, WH, HG and MG advised on appropriate statistical design. SW and WH carried out the statistical analysis for the study. PP supported SW in preparing the paper for publication, including performing the literature search and drafting parts of the manuscript. SW, HG, PP, EM, JB, AB, KB, BC, FLH, JRH, HH, SL, PEP, WR, CR, FAS, DS, JW, MG and WH contributed to study design, reporting and review of the paper in Steering Committee meetings and reviewed the paper prior to submission.

Funding Statement

This study is funded by the National Institute for Health and Care Research (NIHR) Cross-programme [HS&DR] COVID-19 [project reference NIHR132243]. The views expressed are those of the authors and not necessarily those of the NIHR or the Department of Health and Social Care. This report is independent research supported by the NIHR ARC North Thames, NIHR ARC Wessex and NIHR ARC West.

For the purpose of open access, the author has applied a Creative Commons Attribution (CC BY) licence to any Author Accepted Manuscript version arising.

Competing interests

JB reports payments from University College London (UCL) for working as a PPI to prepare content for the DHI since May 2020. KB's research portfolio is part funded by NIHR Applied Research Collaboration Wessex. HG reports working as a Clinical Safety Officer for *Living With*. JRH reports receiving personal fees and fees to institution for honorariums and consultancy payments from AstraZeneca, Boehringer Ingelheim, Chiesi, GlaxoSmithKline, Takeda. JRH also reports receiving sponsorship for attending meetings from AstraZeneca and GlaxoSmithKline. HH reports payment from University of East London for providing a lecture on Long COVID and COVID Recovery in February 2021. SL reports grants from NIHR in which the payment was made to Camden and Islington NHS Trust between the period of October to September 2022. PEP reports grants from the Medical Research Council (MRC) and NIHR outside the submitted work. All other authors declare no competing interests.

For peer review only

Transparency declaration: Sarah Walker affirms that the manuscript is an honest, accurate, and transparent account of the study being reported; that no important aspects of the study have been omitted; and that any discrepancies from the study as planned have been explained.

Data sharing: To request access to the underlying research data, please contact Dr Henry Goodfellow henry.goodfellow.12@ucl.ac.uk.

Ethics

Ethical approval obtained from East Midlands – Derby Research Ethics Committee (reference 288199).

For peer review only

REFERENCES

1. National Institute for Health and Care Excellence. COVID-19 rapid guideline: managing the long-term effects of COVID-19 2022 [updated March 03. Available from: https://www.nice.org.uk/guidance/ng188.
2. World Health Organization. WHO Coronavirus (COVID-19) Dashboard 2022 [updated 2 Aug 2022; cited 2022 Aug 2]. Available from: https://covid19.who.int/ accessed 2 Aug 2022.
3. Maxwell E, Poole R. NIHR Themed Review: Living with Covid19 - Second review: National Institute of Health and Care Research; 2021 [updated 16 March. Available from: https://evidence.nihr.ac.uk/themedreview/living-with-covid19-second-review/ accessed 14 April 2022.
4. Whitaker M, Elliott J, Chadeau-Hyam M, et al. Persistent symptoms following SARS-CoV-2 infection in a random community sample of 508,707 people *medRxiv 21259452 [Preprint]* July 03, 2021 [cited 2022 Apr 14] doi: https://doi.org/10.1101/2021.06.28.21259452
5. Thompson EJ, Williams DM, Walker AJ, et al. Long COVID burden and risk factors in 10 UK longitudinal studies and electronic health records. *Nature Communications* 2022;13(1):3528. doi: 10.1038/s41467-022-30836-0
6. Office for National Statistics. Prevalence of ongoing symptoms following coronavirus (COVID-19) infection in the UK: 7 July 2022 [Available from: https://www.ons.gov.uk/peoplepopulationandcommunity/healthandsocialcare/conditionsanddiseases/bulletins/prevalenceofongoingsymptomsfollowingcoronaviruscovid19infectionintheuk/7july2022 accessed 18 July 2022.
7. Davis HE, Assaf GS, McCorkell L, et al. Characterizing long COVID in an international cohort: 7 months of symptoms and their impact. *EClinicalMedicine* 2021;38:101019. doi: 10.1016/j.eclinm.2021.101019 [published Online First: 20210715]
8. Boscolo-Rizzo P, Guida F, Polesel J, et al. Sequelae in adults at 12 months after mild-to-moderatecoronavirus disease 2019 (COVID-19). *Int Forum Allergy Rhinol* 2021;11:1685-88. doi: 10.1101/2021.04.12.21255343
9. Thompson EJ, Williams DM, Walker AJ, et al. Risk factors for Long-COVID : analyses of 10 longitudinal studies and electronic health records in the UK. *medRxiv 21259277 [Preprint]* July 10, 2021 [cited Nov 21, 2021] doi: 10.1101/2021.06.24.21259277
10. Sandler CX, Wyller VBB, Moss-Morris R, et al. Long COVID and Post-infective Fatigue Syndrome: A Review. *Open Forum Infectious Diseases* 2021;8(10) doi: 10.1093/ofid/ofab440
11. Sivan M, Greenhalgh T, Milne R, et al. Are vaccines a potential treatment for long covid? *BMJ* 2022;377:o988. doi: 10.1136/bmj.o988
12. Torjesen I. Covid-19 patients discharged from hospital have "substantially higher risk" of adverse outcomes and need monitoring. *BMJ* 2022;376:o265. doi: 10.1136/bmj.o265
13. Wise J. Covid-19: Long covid risk is lower with omicron than delta, researchers find. *BMJ* 2022;377:o1500. doi: 10.1136/bmj.o1500
14. Zimmermann P, Pittet LF, Curtis N. Long covid in children and adolescents. *BMJ* 2022;376:o143. doi: 10.1136/bmj.o143
15. Jones R, Davis A, Stanley B, et al. Risk Predictors and Symptom Features of Long COVID Within a Broad Primary Care Patient Population Including Both Tested and Untested Patients. *Pragmat Obs Res* 2021;12:93-104. doi: 10.2147/POR.S316186 [published Online First: 20210811]
16. Sudre CH, Murray B, Varsavsky T, et al. Attributes and predictors of long COVID. *Nat Med* 2021;27(4):626-31. doi: 10.1038/s41591-021-01292-y [published Online First: 2021/03/12]
17. Waters T, Wernham T. Long COVID and the labour market: The Institute for Fiscal Studies, 2022.
18. NHS England and NHS Improvement. Long-COVID: the NHS plan for 2021/22 2021 [updated June. Available from: https://www.england.nhs.uk/coronavirus/wp-content/uploads/sites/52/2021/06/C1312-long-covid-plan-june-2021.pdf accessed 21 November 2022.

For peer review only

19. Thorlby R, Gardner T, Allen L, et al. The NHS Long Term Plan and COVID-19: Assessing progress and the pandemic's impact. London: The Health Foundation, 2021.
20. Murray E, Goodfellow H, Bindman J, et al. Development, deployment and evaluation of digitally enabled, remote, supported rehabilitation for people with long COVID-19 (Living With COVID-19 Recovery): protocol for a mixed-methods study. *BMJ Open* 2022;12(2):1-9. doi: 10.1136/bmjopen-2021-057408 [published Online First: 20220207]
21. Mundt JC MA, Shear MK, Greist JH. The Work and Social Adjustment Scale : a simple measure of impairment in functioning. *Br J Psychiatry* 2002;180(46):61-4. doi: 10.1192/bjp.180.5.461
22. EuroQol Research Foundation. EQ-5D-5L Userguide, 2019.
23. Ministry of Housing CaLG. English indices of deprivation 2019: Research report, 2019.
24. Yorke J, Moosavi SH, Shuldham C, et al. Quantification of dyspnoea using descriptors: development and initial testing of the Dyspnoea-12. *Thorax* 2010;65(1):21-6. doi: 10.1136/thx.2009.118521 [published Online First: 20091208]
25. Montan I, Löwe B, Cella D, et al. General Population Norms for the Functional Assessment of Chronic Illness Therapy (FACIT)-Fatigue Scale. *Value Health* 2018;21(11):1313-21. doi: 10.1016/j.jval.2018.03.013 [published Online First: 2018/11/18]
26. Piper BF, Cella D. Cancer-related fatigue: Definitions and clinical subtypes. *J Natl Compr Cancer Netw* 2010;8(8):958-66. doi: 10.6004/jnccn.2010.0070
27. Bestall JC PE, Garrod R, Garnham R, Jones PW, Wedzicha JA. Usefulness of the Medical Research Council (MRC) dyspnoea scale as a measure of disability in patients with chronic obstructive pulmonary disease. *Thorax* 1999;54:581-6. doi: 10.1136/thx.54.7.581
28. Horwitz LI, Garry K, Prete AM, et al. Six-Month Outcomes in Patients Hospitalized with Severe COVID-19. *J Gen Intern Med* 2021;36(12):3772-77. doi: 10.1007/s11606-021-07032-9 [published Online First: 20210805]
29. Paladini L, Hodder R, Cecchini I, et al. The MRC dyspnoea scale by telephone interview to monitor health status in elderly COPD patients. *Respir Med* 2010;104(7):1027-34. doi: 10.1016/j.rmed.2009.12.012
30. Cella D LJ, Chang CH, Peterman A, Slavin M. Fatigue in cancer patients compared with fatigue in the general United States population. *Cancer* 2002;94:528-38. doi: https://doi.org/10.1002/cncr.10245
31. Spitzer RL, Kroenke K, Williams JBW, et al. A Brief Measure for Assessing Generalized Anxiety Disorder The GAD-7. *Arch Intern Med* 2006;166(10):1092-7. doi: 10.1001/archinte.166.10.1092
32. Sullivan M, Edgley K, DeHousx E. A survey of multiple sclerosis, part 1: perceived cognitive problems and compensatory strategy use. *Can J Rehabil* 1990;4(2):99-105.
33. Julian LJ, Yazdany J, Trupin L, et al. Validity of brief screening tools for cognitive impairment in rheumatoid arthritis and systemic lupus erythematosus. *Arthritis Care Res (Hoboken)* 2012;64(3):448-54. doi: 10.1002/acr.21566
34. Lam R. Subjective measures of cognitive dysfunction in major depressive disorder. In: McIntyre RS, ed. Cognitive Impairment in Major Depressive Disorder: Clinical Relevance, Biological Substrates, and Treatment Opportunities. Cambridge: Cambridge University Press 2016:242-50.
35. Kroenke K, Strine TW, Spitzer RL, et al. The PHQ-8 as a measure of current depression in the general population. *J Affect Disord* 2009;114(1-3):163-73. doi: 10.1016/j.jad.2008.06.026 [published Online First: 20080827]
36. Kroenke K, Spitzer R. The PHQ-9: a new depression and diagnostic severity measure. *Psychiatry* 2002;32(9):509-15. doi: https://doi.org/10.3928/0048-5713-20020901-06
37. McKelvey RD, Zavoina W. A statistical model for the analysis of ordinal level dependent variables. *J Math Sociol* 1975;4(1):103-20. doi: 10.1080/0022250X.1975.9989847

38. Zahra D, Qureshi A, Henley W, et al. The work and social adjustment scale: Reliability, sensitivity and value. *International Journal of Psychiatry in Clinical Practice* 2014;18(2):131-38. doi: 10.3109/13651501.2014.894072
39. Hommel M, Trabucco-Miguel, S, Joray, S, Naegele, B, Gonnet, N, Jaillard, A,. Social dysfunctioning after mild to moderate first-ever stroke at vocational age. *Journal of Neurology, Neurosurgery & Psychiatry* 2009;80(4):371-75. doi: 10.1136/jnnp.2008.157875.
40. Paracha N, Abdulla A, MacGilchrist KS. Systematic review of health state utility values in metastatic non-small cell lung cancer with a focus on previously treated patients. *Health Qual Life Outcomes* 2018;16(179) doi: 10.1186/s12955-018-0994-8
41. Pourrahmat MM, Kim A, Kansal AR, et al. Health state utility values by cancer stage: a systematic literature review. *Eur J Health Econ* 2021;22:1275-88. doi: 10.1007/s10198-021-01335-8
42. Acaster S, Dickerhoof R, DeBusk K, et al. Qualitative and quantitative validation of the FACIT-fatigue scale in iron deficiency anemia. *Health Qual Life Outcomes* 2015;13:60. doi: 10.1186/s12955-015-0257-x [published Online First: 2015/05/20]
43. Chen K, Marsh EB. Chronic post-stroke fatigue: It may no longer be about the stroke itself. *Clin Neurol Neurosurg* 2018;174:192-97. doi: https://doi.org/10.1016/j.clineuro.2018.09.027
44. Wang S-Y, Zang X-Y, Fu S-H, et al. Factors related to fatigue in Chinese patients with end-stage renal disease receiving maintenance hemodialysis: a multi-center cross-sectional study. *Ren Fail* 2015;38(3):442-50. doi: 10.3109/0886022X.2016.1138819
45. Christensen KR, Ainsworth MA, Steenholdt C, et al. Fatigue is a systemic extraintestinal disease manifestation largely independent of disease activity, chronicity, and nutritional deficiencies in inflammatory bowel disease on biologics. *Scand J Gastroenterol* 2022;57(9):1051-57. doi: 10.1080/00365521.2022.2060049
46. Evans RA, McAuley H, Harrison EM, et al. PHOSP-COVID Collaborative Group. Physical, cognitive, and mental health impacts of COVID-19 after hospitalisation (PHOSP-COVID): a UK multicentre, prospective cohort study. *Lancet Respir Med* 2021;9(11):1275-87. doi: 10.1016/S2213-2600(21)00383-0 [published Online First: 2021 Oct 7]
47. Heightman M, Prashar J, Hillman TE, et al. Post-COVID-19 assessment in a specialist clinical service: a 12-month, single-centre, prospective study in 1325 individuals. *BMJ Open Respiratory Research* 2021;8:e001041. doi: https://doi.org/10.1136/bmjresp-2021-001041
48. International Consortium for Health Outcomes Measurement (ICHOM). Sets of Patient-Centered Outcome Measures [Available from: https://connect.ichom.org/patient-centered-outcome-measures/ accessed 5th July 2022.
49. Munblit D, Nicholson T, Akrami A, et al. A core outcome set for post-COVID-19 condition in adults for use in clinical practice and research: an international Delphi consensus study. *Lancet Respir Med* 2022;10(7):715-24. doi: 10.1016/S2213-2600(22)00169-2.
50. Taquet M, Dercon Q, Luciano S, et al. Incidence, co-occurrence, and evolution of long-COVID features: A 6-month retrospective cohort study of 273,618 survivors of COVID-19. *PLoS Med* 2021;18(9):1-22. doi: http://dx.doi.org/10.1371/journal.pmed.1003773 [published Online First: 28 September 2021]
51. Jakobsen JC, Gluud C, Wetterslev J, et al. When and how should multiple imputation be used for handling missing data in randomised clinical trials – a practical guide with flowcharts. *BMC Medical Research Methodology* 2017;17(1):162. doi: 10.1186/s12874-017-0442-1
52. Devine BF, Foley N, Ward M. Women and Economy: House of Commons Library, 2021.
53. The Department of Health and Social Care (DHSC) and Office for National Statistics (ONS). Direct and Indirect Health Impacts of COVID-19 in England: The Department of Health and Social Care (DHSC) and Office for National Statistics (ONS), 2021.

For peer review only

Figures Legends:

Figure 1a: Heat Map showing the distribution of each patient's (n=2502) WSAS scores (higher score representing an increase in functional limitations) compared to their corresponding fatigue levels FACIT-F (reversed scale) and depression (PHQ-8) levels. The dashed line represents the threshold values for significant fatigue on the x-axis and clinical depression on the y-axis.

Figure 1b: Heat Map showing the distribution of each patient's (n=2520) WSAS scores (higher score representing an increase in functional limitations) compared to their corresponding fatigue levels (FACIT-F (reversed scale) and brain fog (PDQ5) levels. The dashed line represents the threshold value for significant fatigue on the x-axis and moderate brain fog on the y-axis.

Figure 2: Change in proportion of variation in WSAS explained (R-squared) when PROMs were removed from the linear regression models for each WSAS domain.

For peer review only

Figure 1a:

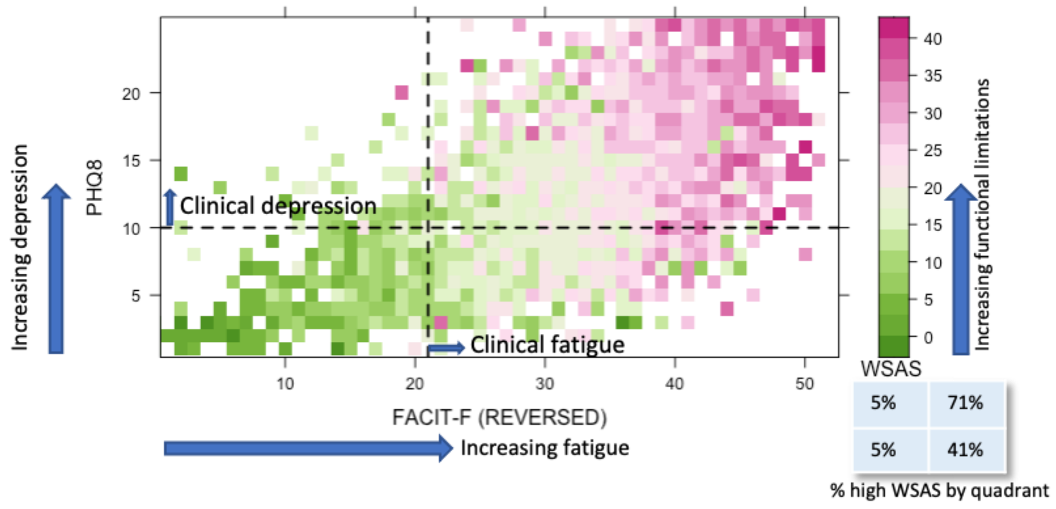

Figure 1b:

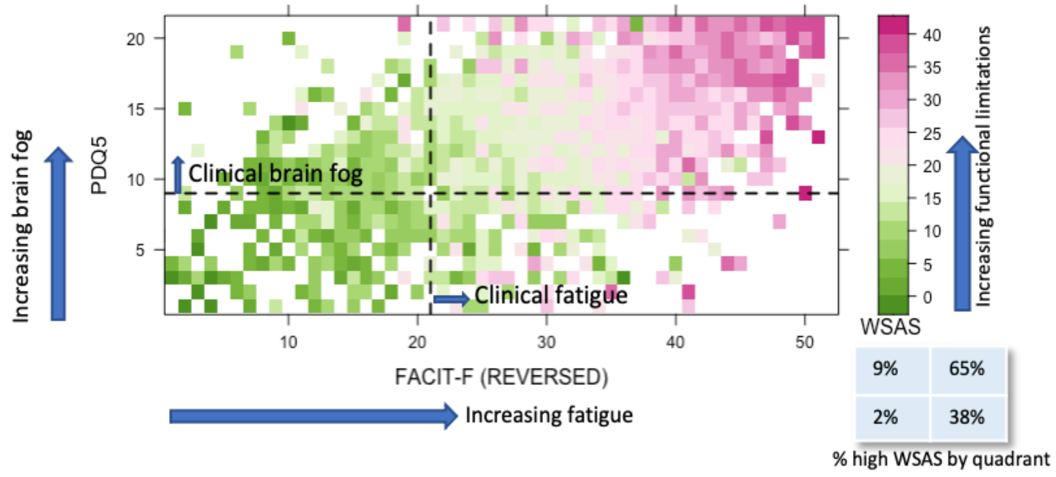

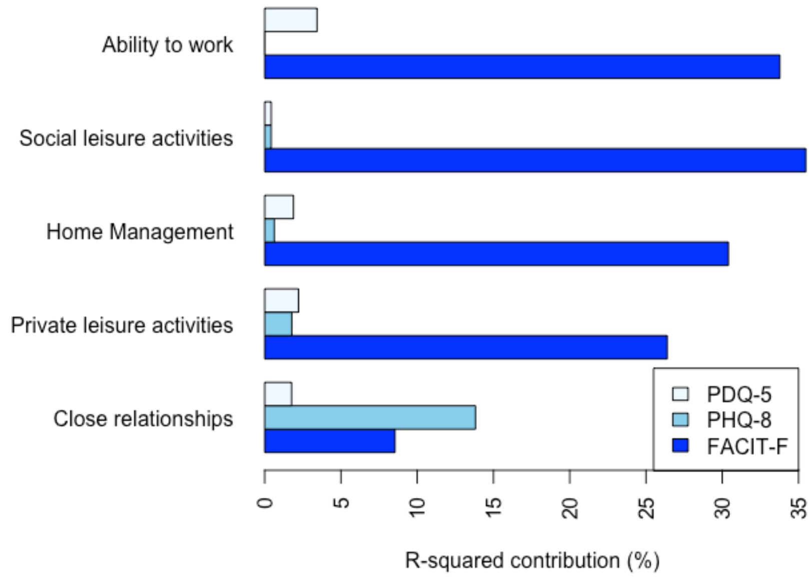
Ability to work
Social leisure activities
Home Management
Private leisure activities
Close relationships
PDQ-5
PHQ-8
FACIT-F
0
5
10
15
20
25
30
35
R-squared contribution (%)

Appendix

Appendix 1: Mean scores for the overall WSAS score and individual WSAS domains

WSAS Domain	N	Mean (SD)	Range and threshold values
WSAS overall score	2627	20.6 (9.9)	Range:0-40<br><10: subclinical<br>10 – 19: significant<br>**>20: Moderately severe**
Ability to work*	2621	4.6 (2.4)	Subscale range: 0-8<br>0: not at all affected to<br>8: very severely affected
Home management	2627	4.2 (2.2)	
Social leisure activities	2627	4.0 (2.2)	
Private leisure activities	2627	4.7 (2.3)	
Close relationships	2627	3.0 (2.4)	

* Reduced number of completed answers as patients who had retired or chose not to work did not need to answer this question.

For peer review only

Appendix 2: EQ-5D-5L frequencies and proportions reported by dimension and level

	Mobility n (%)	Self-care n (%)	Usual activities n (%)	Pain / discomfort n (%)	Anxiety / depression n (%)
**Level 1** (No problems)	712(26.9)	318(12.0)	959(36.3)	101(3.8)	134(5.1)
**Level 2** (Slight problems)	795(30.1)	1702(64.4)	250(9.5)	983(37.2)	701(26.5)
**Level 3** (Moderate problems)	309(11.7)	98(3.7)	506(19.1)	358(13.5)	675(25.5)
**Level 4** (Severe problems)	810(30.6)	511(19.3)	759(28.7)	373(14.1)	267(10.1)
**Level 5** (Extreme problems / unable to do)	17(0.6)	14(0.5)	169(6.4)	828(31.3)	866(32.8)
Total	2643(100)	2643(100)	2643(100)	2643(100)	2643(100)

For peer review only

Appendix 3: Working days lost due to Post-COVID syndrome in 28 days prior to completion of Service Use Questionnaire

Number completed Service Use questionnaire	2600
Number (%) who lost 1 or more days from work	1321 (50.8)
Mean number of working days lost (SD)*	13.8 (10.7)
Median number of working days lost (IQR)*	10 (4 to 28)

* in those who lost 1 or more days off work

Appendix 4: EQ-5D index score multivariable model for different patient characteristics and PROM scores (N=2405)

Patient Characteristics		Model coefficients (95% CI)	p-value	Change in R-squared * [Full model R-sq=0.573)	Standardised effect size
Age	18 to 29	Reference			
	30 to 39	-0.02 (-0.05, 0.01)	0.219		
	40 to 49	-0.03 (-0.06, -0.01)	0.009		
	50 to 59	-0.03 (-0.06, -0.01)	0.018		
	60 to 69	-0.06 (-0.09, -0.03)	<0.0001		
	70 and over	-0.07 (-0.12, -0.02)	0.005		
Gender	Male	Reference			
	Female	0.00 (-0.01, 0.02)	0.786		
Educational	No education	Reference			

For peer review only

level	School leaver (NVQ 1-2)	0.00 (-0.04, 0.04)	0.948		
	A-Level (NVQ-3)	0.02 (-0.02, 0.05)	0.389		
	Degree (NVQ-4)	0.01 (-0.02, 0.05)	0.464		
	Postgraduate degree (NVQ-5)	0.02 (-0.01, 0.06)	0.210		
Ethnicity	White	Reference			
	Non-white	-0.02 (-0.04, 0.00)	0.073		
IMD Quintile	1 (most deprived)	Reference			
	2	0.02 (0.00, 0.05)	0.059		
	3	0.03 (0.00, 0.05)	0.025		
	4	0.05 (0.02, 0.07)	<0.0001		
	5 (least deprived)	0.03 (0.01, 0.06)	0.008		
PROMs	FACIT-Fatigue (reversed scale)	-0.01 (-0.01, -0.01)	<0.0001	0.048	-0.080
	PHQ-8	-0.01 (-0.01, -0.01)	<0.0001	0.010	-0.044

For peer review only

	GAD-7	-0.01 (-0.01, -0.01)	<0.0001	0.018	-0.051
	MRC Dyspnoea Scale: Grade 1	Reference		0.032	
	MRC Dyspnoea Scale: Grade 2	0.02 (0.00, 0.04)	0.108		0.010
	MRC Dyspnoea Scale: Grade 3	-0.02 (-0.04, 0.01)	0.191		-0.009
	MRC Dyspnoea Scale: Grade 4	-0.08 (-0.11, -0.05)	<0.0001		-0.030
	MRC Dyspnoea Scale: Grade 5	-0.25 (-0.30, -0.20)	<0.0001		-0.045
	Dyspnoea-12	0.00 (0.00, 0.00)	<0.0001	0.003	-0.020

* Reduction in R-squared value when variable is removed from the final model. Overall model has R-squared value of 0.573

For peer review only

Appendix Figure 1: Frequency distribution of the first reported (baseline) WSAS

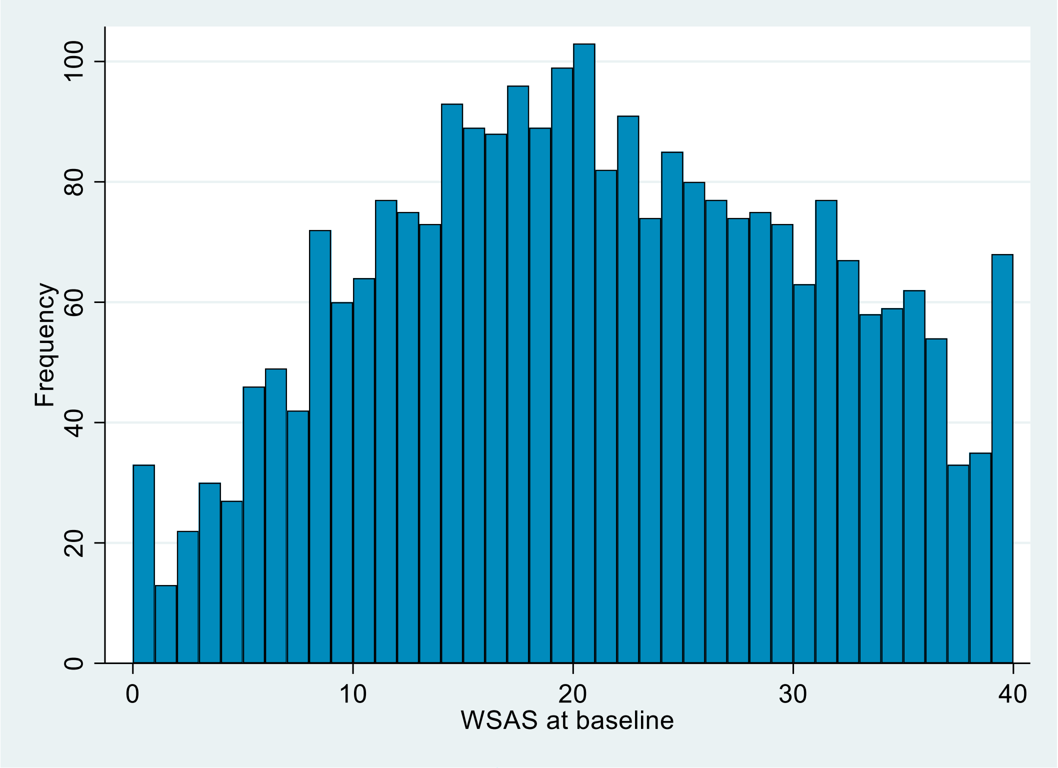

Appendix Figure 2: Frequency distribution of the first reported (baseline) EQ-5D Index Score

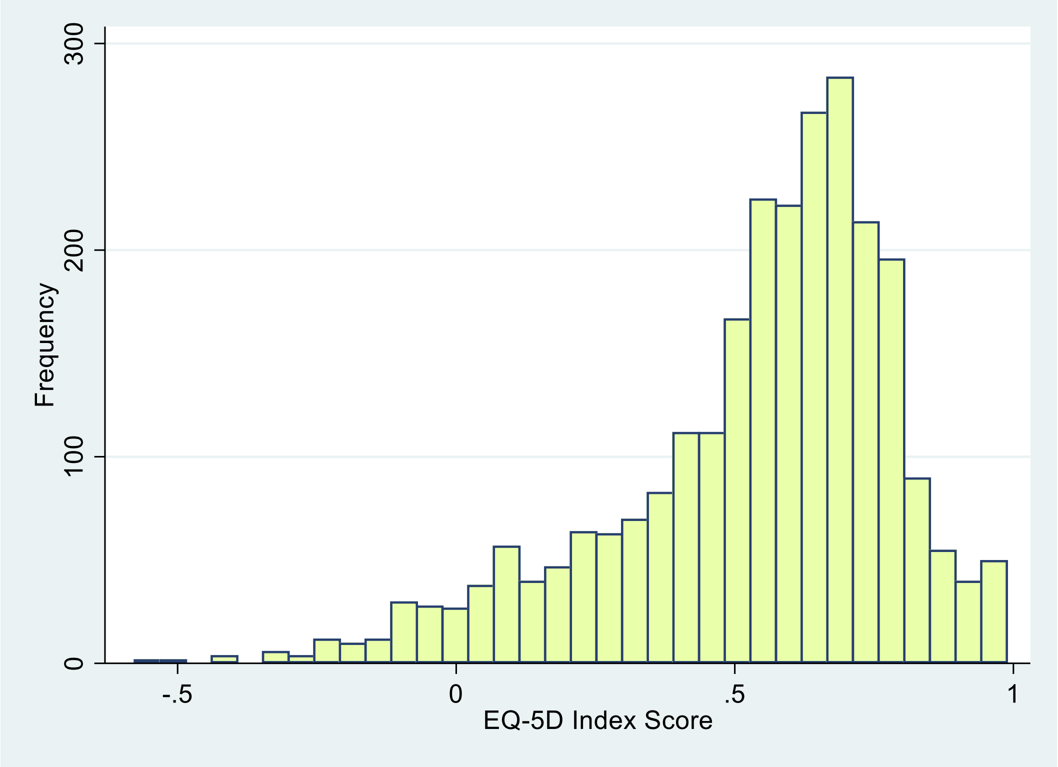

STROBE Statement—Checklist of items that should be included in reports of ***cross-sectional studies***

	Item No	Recommendation	Page No
**Title and abstract**	1	(*a*) Indicate the study's design with a commonly used term in the title or the abstract	1
		(*b*) Provide in the abstract an informative and balanced summary of what was done and what was found	3
**Introduction**			
Background/rationale	2	Explain the scientific background and rationale for the investigation being reported	5
Objectives	3	State specific objectives, including any prespecified hypotheses	6
**Methods**			
Study design	4	Present key elements of study design early in the paper	6
Setting	5	Describe the setting, locations, and relevant dates, including periods of recruitment, exposure, follow-up, and data collection	6-7
Participants	6	(*a*) Give the eligibility criteria, and the sources and methods of selection of participants	7
Variables	7	Clearly define all outcomes, exposures, predictors, potential confounders, and effect modifiers. Give diagnostic criteria, if applicable	7-8
Data sources/ measurement	8*	For each variable of interest, give sources of data and details of methods of assessment (measurement). Describe comparability of assessment methods if there is more than one group	8
Bias	9	Describe any efforts to address potential sources of bias	10
Study size	10	Explain how the study size was arrived at	7
Quantitative variables	11	Explain how quantitative variables were handled in the analyses. If applicable, describe which groupings were chosen and why	9-10
Statistical methods	12	(*a*) Describe all statistical methods, including those used to control for confounding	9-10
		(*b*) Describe any methods used to examine subgroups and interactions	N/A
		(*c*) Explain how missing data were addressed	10
		(*d*) If applicable, describe analytical methods taking account of sampling strategy	N/A
		(*e*) Describe any sensitivity analyses	N/A
**Results**			
Participants	13*	(a) Report numbers of individuals at each stage of study—eg numbers potentially eligible, examined for eligibility, confirmed eligible, included in the study, completing follow-up, and analysed	11
		(b) Give reasons for non-participation at each stage	N/A
		(c) Consider use of a flow diagram	N/A
Descriptive data	14*	(a) Give characteristics of study participants (eg demographic, clinical, social) and information on exposures and potential confounders	11-17
		(b) Indicate number of participants with missing data for each variable of interest	15-16
Outcome data	15*	Report numbers of outcome events or summary measures	15-16

For peer review only

Main results	16	(*a*) Give unadjusted estimates and, if applicable, confounder-adjusted estimates and their precision (eg, 95% confidence interval). Make clear which confounders were adjusted for and why they were included	19-20
		(*b*) Report category boundaries when continuous variables were categorized	15-16
		(*c*) If relevant, consider translating estimates of relative risk into absolute risk for a meaningful time period	19-20
Other analyses	17	Report other analyses done—eg analyses of subgroups and interactions, and sensitivity analyses	N/A
**Discussion**			
Key results	18	Summarise key results with reference to study objectives	23
Limitations	19	Discuss limitations of the study, taking into account sources of potential bias or imprecision. Discuss both direction and magnitude of any potential bias	24
Interpretation	20	Give a cautious overall interpretation of results considering objectives, limitations, multiplicity of analyses, results from similar studies, and other relevant evidence	24-25
Generalisability	21	Discuss the generalisability (external validity) of the study results	25
**Other information**			
Funding	22	Give the source of funding and the role of the funders for the present study and, if applicable, for the original study on which the present article is based	25

*Give information separately for exposed and unexposed groups.

Note: An Explanation and Elaboration article discusses each checklist item and gives methodological background and published examples of transparent reporting. The STROBE checklist is best used in conjunction with this article (freely available on the Web sites of PLoS Medicine at http://www.plosmedicine.org/, Annals of Internal Medicine at http://www.annals.org/, and Epidemiology at http://www.epidem.com/). Information on the STROBE Initiative is available at www.strobe-statement.org.

For peer review only

For peer review only - http://bmjopen.bmj.com/site/about/guidelines.xhtml
